# Single cell cloning and recombinant monoclonal antibodies generation from RA synovial B cells reveal frequent targeting of citrullinated histones of NETs

**DOI:** 10.1136/annrheumdis-2015-208356

**Published:** 2015-12-09

**Authors:** Elisa Corsiero, Michele Bombardieri, Emanuela Carlotti, Federico Pratesi, William Robinson, Paola Migliorini, Costantino Pitzalis

**Affiliations:** 1 Centre for Experimental Medicine & Rheumatology, William Harvey Research Institute, Barts and The London School of Medicine & Dentistry, Queen Mary University of London, London, UK; 2 Clinical Immunology and Allergy Unit, Department of Clinical & Experimental Medicine, University of Pisa, Pisa, Italy; 3 Stanford University School of Medicine, Stanford, USA

**Keywords:** Rheumatoid Arthritis, B cells, Autoantibodies

## Abstract

**Objectives:**

Rheumatoid arthritis (RA) is characterised by breach of self-tolerance towards citrullinated antigens with generation of anti-citrullinated peptide/proteins antibodies (ACPA). Currently, the nature and source of citrullinated antigens driving the humoral autoimmune response within synovial ectopic lymphoid structures (ELS) is a crucial unknown aspect of RA pathogenesis. Here we characterised the autoreactive B-cell response of lesional B cells isolated from ELS+RA synovium.

**Methods:**

Single synovial tissue CD19+cells were Fluorescence Activated Cell Sorting (FACS)-sorted and V_H_/V_L_ Ig genes cloned to generate recombinant monoclonal antibodies (rmAbs) from patients with ELS+/ACPA+RA.

**Results:**

RA-rmAbs immunoreactivity analysis provided the following key findings: (1) in a chIP-based array containing 300 autoantigens and in a ‘citrullinome’ multiplex assay, a strong reactivity against citrullinated histones H2A/H2B (citH2A/H2B) was observed in ∼40% of RA-rmAbs, followed by cit-fibrinogen and cit-vimentin; (2) anti-citH2A/H2B-reactive RA-rmAbs (but not anti-citH2A/H2B negative) selectively recognised neutrophil extracellular traps (NETs) from peripheral blood and/or RA joint neutrophils; (3) anti-citH2A/citH2B and anti-NET immunobinding was dependent on affinity maturation and was completely abrogated following reversion of hypermutated IgV_H_/V_L_ genes to germline sequences; (4) ELS+ (not ELS−) RA synovial tissues engrafted into Severe Combined ImmunoDeficiency (SCID) mice released human anti-citH2A/citH2B and anti-NET antibodies in association with the intra-graft expression of CXCL13 and lymphotoxin (LT)-β, two master regulators of ELS.

**Conclusion:**

We provided novel evidence that B cells differentiated within synovial ELS in the RA joints frequent target deiminated proteins which could be generated during NETosis of RA synovial neutrophils including histones. Thus, NETs could represent a source of citrullinated antigens fuelling the ACPA autoimmune response within the RA synovium.

## Introduction

Rheumatoid arthritis (RA) is characterised by breach of self-tolerance towards citrullinated proteins (anti-citrullinated peptide/proteins antibodies (ACPA)), which can occur years prior to clinical onset of RA at extra-articular sites.^1–6^ Several post-translationally deiminated proteins have been indicated as a potential source of citrullinated antigens in the RA joints,^3^ but to date their cellular source and specific contribution to the lesional ACPA response is unknown.

Around 40%–50% of patients with RA display synovial ectopic lymphoid structures (ELS) characterised by B-cell follicles supporting a germinal centre (GC) response.^7^ ^8^ Synovial ELS are self-sustained niches whereby autoreactive B cells undergo antigen-driven selection/differentiation with local antibody diversification through Ig genes somatic hypermutation (SHM)^9^ and class switching.^10^

Citrullination, or arginine deimination, is catalysed by the enzyme peptidyl-arginine-deiminase (PAD). In the RA synovium, monocyte–macrophages are the main source of this enzyme.^11^ ^12^ As a result, citrullination of fibrinogen, vimentin and α-enolase, among others, has been observed within the RA joints and associated with circulating ACPA.^13–15^ Accordingly, monoclonal antibodies generated from synovial fluid B cells frequently react against citrullinated antigens.^16^

PAD-mediated deimination of core histones (H2A/H2B/H3/H4) has been described in neutrophils during the neutrophil extracellular traps (NETs) formation, or NETosis, a form of cell death which enhances the antimicrobial properties of activated neutrophils.^17^ ^18^ Interestingly, RA synovial fluid neutrophils display an enhanced NETosis in the absence of microbial stimuli due to the RA proinflammatory milieu^19^ and RA sera react against citrullinated H4 from NETs.^2^

At present, direct evidence that synovial B cells from ELS+RA recognise citrullinated proteins and the specific contribution of different citrullinated antigens in fuelling the lesional ACPA production is missing. To this aim, we investigated the immunoreactivity of recombinant monoclonal antibodies (rmAbs) generated from single synovial B-cell clones obtained from patients with ELS+/ACPA+RA.

## Materials and methods

A full list of methods is reported in the online supplementary methods.

### Patients

Three synovial tissues from total joint replacement were obtained after informed consent (National-Research-Ethics-Service–Committee-London-LREC05/Q0703/198) from patients with ACPA+ RA (all females, age range 66–75, all on combination Disease-Modifying AntiRheumatic Drug (DMARD) therapy including methotrexate) diagnosed according to the revised American College of Rheumatology (ACR) criteria.^20^ This board specifically approved the collection of the synovial tissue. Synovial tissue was dissected and processed as previously described.^10^

### Synovial mononuclear cell isolation and CD19+ cell FACS sorting

Mononuclear cells were isolated from fresh synovial tissue specimens obtained as above. Detailed method is reported in the online supplementary methods.

### Generation of recombinant monoclonal antibodies

Single-cell real time-PCR reactions and IgV gene amplification were performed as described in refs. 21 and 22. Briefly, cDNA from CD3-CD19+B cells was amplified using reverse primers that bind the Cμ/Cγ or Cα constant region in three independent nested-PCR. The complete sequence of primers is reported in online supplementary table S1. Aliquots of Variable Heavy (VH)/Vκ/Vλ chains second PCR products were sequenced with the respective reverse primer and analysed by IgBlast. IgH complementary determining region (CDR)3 amino acids and length were determined as described.^21^ The V gene somatic mutations analysis was performed using IMmunoGeneTics/Variable (IMGT/V)-QUEry and STandardization (QUEST) to characterise the silent versus non-silent mutation in each Framework Region (FR)/CDR region to determine the R:S ratio. The expression vector cloning strategy and the monoclonal antibody production were performed as described in ref. 21. Immunoglobulin Analysis Tool (IgAT) software was used to calculate the probability of antigen-driven selection within the Ig repertoire of the RA-rmAbs,^23^ as previously described.^22^

### Multiplex autoantibody assay

The multiplex autoantibodies assay containing 20 citrullinated RA-associated antigens (see online supplementary table S2) was performed as previously published.^5^ Briefly, rmAbs were added at 10 μg/mL to custom Bio-Plex beads associated with RA putative autoantigens and incubated at room temperature (RT) for 1 h. After washing, PhycoErythrin (PE)-anti-human-IgG antibody was added to the beads and incubated at RT. The fluorescence of PE detected (Luminex200,Bio-Plex-Software V.5.0, Bio-Rad) reflects the amount of antibodies that bind to the beads.

### ELISA assay for anti-citrullinated H2A and H2B

ELISA plates were coated with citrullinated or unmodified histones H2A or H2B at 10 μg/mL in Phosphate Buffered Saline (PBS). Samples at 10 μg/mL were transferred into the ELISA plate and incubated for 2 h (SCID serum diluted 1:10). Unbound antibodies were removed by washing before incubation for 1 h with horseradish peroxidase coupled goat-anti-human-IgG. Assays were developed using TetraMethylBenzidine (TMB) Substrate Reagent Set (Becton Dickinson Optical Enzyme ImunoAssay (BDOptEIA)). Optical densities (ODs) were measured at 450 nm.

### Stimulation of NETosis and immunofluorescence microscopy on NETs

Neutrophils were isolated from peripheral blood (PB) of healthy donors or synovial fluid of two patients with RA using discontinuous gradient centrifugation according to ref. 24 and seeded onto cell culture cover slides at 2×10^5^ cells/well. Cells were either fixed with 4% paraformaldehyde or before fixation activated with 100 nM Phorbol Myristate Acetate (PMA) for 4 h at 37°C. NETs were stained with RA-rmAbs or Sjögren's syndrome (SS) control rmAbs diluted in PBS for 1 h (RT). After washing with Tris Buffered Saline (TBS), Alexa488-goat-anti-human-IgG (Invitrogen, 1:200) was added for 30 min (RT). 4', 6-DiAmidino-2-PhenylIndole (DAPI) (Invitrogen) was added to visualise the NETs. All sections were visualised using an Olympus BX60 microscope. All rmAbs have been tested at 10 μg/mL.

### RA synovial tissue transplantation into SCID mice

RA SCID chimaeras were established as previously described.^10^ Sera and synovial grafts were harvested and analysed for autoantibodies and gene expression profiling, respectively, as previously reported.^10^ Detailed method is reported in the online supplementary methods.

### Statistical analysis

Differences in quantitative variables were analysed by the Mann–Whitney U test (two groups) and by the Kruskal–Wallis with Dunn's post-test (multiple groups). χ^2^ test with Yates’s correction or Fisher's exact test when required were used to evaluate associations of qualitative variables in the different groups. All the statistical analyses were performed using GraphPad-Prism5.01. A p value <0.05 was considered statistically significant. For array reactivity, the Significance Analysis of Microarrays algorithm to normalise array median fluorescence intensity values was applied.

## Results

### Ig gene analysis demonstrates intra-synovial antigen-driven B-cell affinity maturation and clonal diversification in ELS+RA synovium

**Figure 1:**
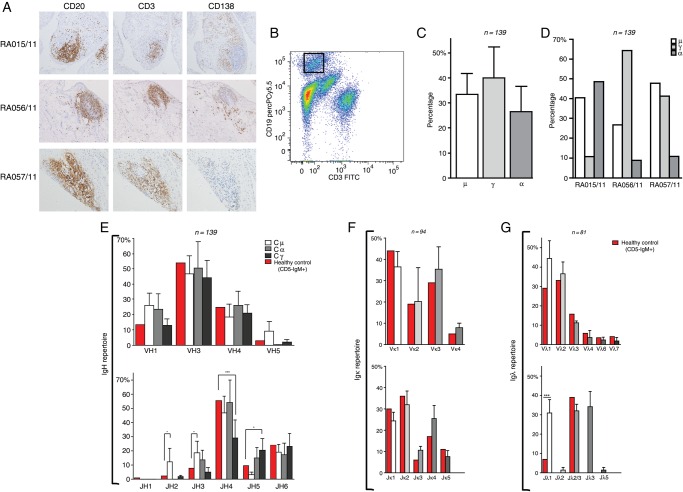
Histological characterisation of synovial ectopic lymphoid structures (ELS), single synovial CD19+ cell sorting and V_H_/V_L_ Ig gene analysis. (A) Representative immunohistological characterisation of synovial tissue samples from patients with rheumatoid arthritis (RA) used in this study (RA015/11, RA056/11 and RA057/11). To assess the presence of ELS, sequential paraffin tissue sections were stained for CD20 (B cells, left-panel), CD3 (T cells, central-panel) and CD138 (plasma cells, right-panel), respectively. (B) Isolation strategy of single CD19+ RA synovial B cells is shown. Mononuclear cells were surface labelled with fluorochrome-coupled anti-CD19 and anti-CD3 antibodies; the sorting gate strategy for single CD19+CD3− B cell is shown. A total of 50 000 events is shown in the FACS plot. (C) The frequencies of μ, γ and α heavy chain among all CD19+ B cells for which VH sequences were obtained are shown. (D) Frequencies of μ, γ and α heavy chain among all CD19+ B cells for each synovial tissue are shown. (E) The VH and JH gene repertoire of single CD19+ synovial B cells for each individual chain isotype, μ (white), α (grey) and γ (black) is shown. (F) The Vκ and (G) Vλ gene repertoire are shown. The red bars indicate the V(H+L) gene repertoire of peripheral blood-naïve B cells. The absolute number of sequences analysed is reported over each graph. Error bars in bar graph indicate SEM for individual patients. p Values compare data from RA synovial B cells with peripheral blood-naïve B cells; *p<0.05; ***p<0.001.

**Figure 2:**
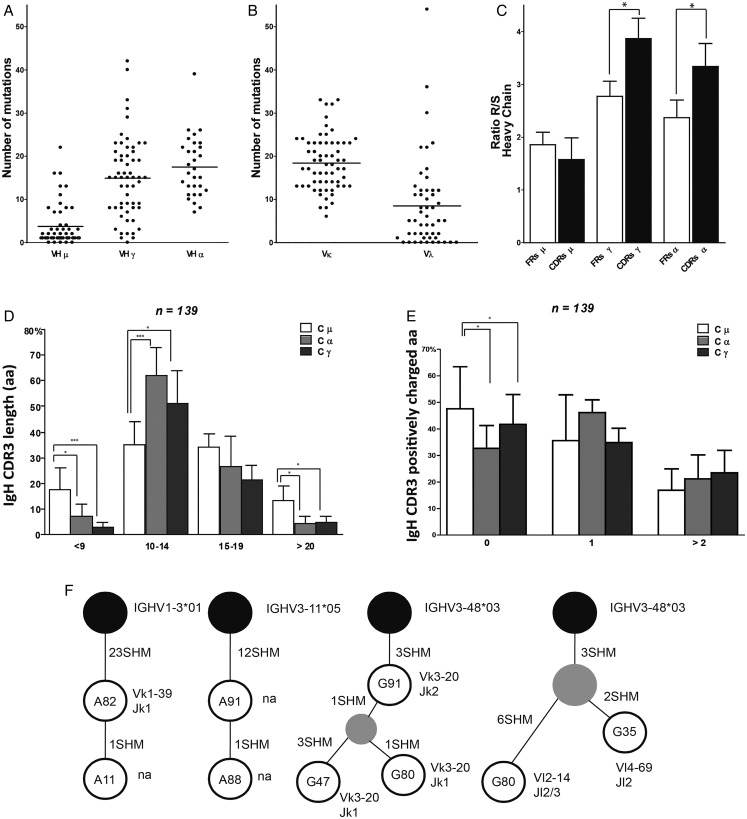
VH/VL Ig gene analysis demonstrating intra-synovial antigen-driven B-cell affinity maturation and clonal diversification. Ig gene sequences of CD19+ synovial B cells were analysed for the absolute numbers of somatic mutations in VH genes (FRs+CDRs) (shown separately for IgM, IgG and IgA clones in (A) as well as VL genes (κ and λ shown separately in (B)). (C) Frequency of replacement (R) and silent (S) mutation ratio in FR (white) and CDR (black) regions for IgM, IgG and IgA is shown. Significant differences between the R/S ratio in FR versus CDR regions of IgG and IgA clones are shown: (D) IgH CDR3 aa length and (E) number of positively charged aa in the CDR3 is shown for each heavy chain isotype separately. (F) Lineage trees generated by comparison of Ig VH sequences of synovial B cells are shown. Clonally related B cells were identified based on cells derived from a single germline rearrangement characterised by the same V, D and J gene usage, CDR3 length and sequence. The synovial B-cell clones are depicted as white circle, the putative common progenitor as grey circle and the germline sequences as black circle. The number inside the circle corresponds to the name of the clone and the number beside the line represents the additional mutation acquired. At the bottom of each clone the correspondent Ig VL sequence is indicated; na=not available and it indicates those genes for the light chain only that could not be amplified; *p<0.05 and **p<0.01.

**Figure 3:**
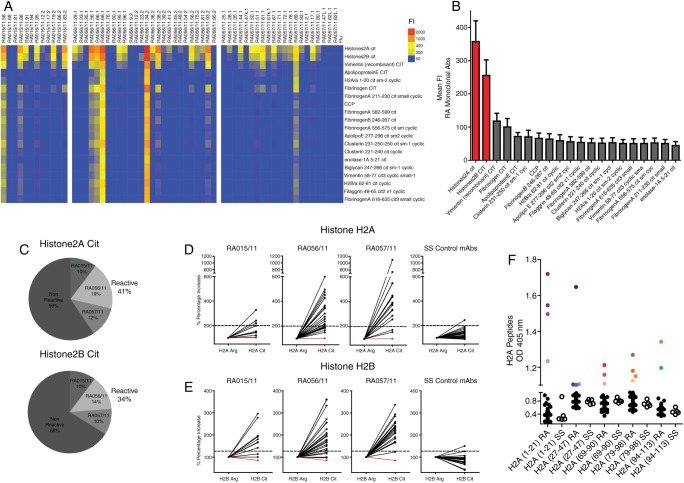
Characterisation of the binding of the rheumatoid arthritis (RA) synovial recombinant monoclonal antibodies (rmAbs) towards citrullinated antigens demonstrates biased immunoreactivity towards citrullinated histones. (A) Luminex heatmap is shown. Heatmap tiles reflect the amount of IgG autoantibody binding reactivity based on the fluorescence intensity scale as indicated on the top-right. rmAb-IDs (individual columns, top labelling) and the location of each citrullinated antigen in the assay (individual rows, right side legend) are shown. (B) Column bar graph representation of the mean fluorescence intensity (FI+SEM) of the luminex heatmap for each citrullinated antigen. (C) Pie charts showing the general percentage of reactivity towards citH2A (top) and citH2B (bottom) histones of the RA-rmAbs after correction of background signal and breakdown of the prevalence in each individual synovial tissue. (D and E) Binding of the RA and control rmAbs (30 naïve/memory B-cell clones from patients with Sjögren's syndrome (SS)) to native and in vitro citrullinated histone H2A and H2B tested by ELISA. Results are grouped according to tissue donors and shown as increased percentage of binding comparing native versus citrullinated histones. A flu control rmAb is shown in red. The dotted horizontal line represents the cut-off for positivity of the rmAbs which was determined as the mean+2SD of the 30 SS rmAbs reactivity (right panel). (F) Binding of the RA-rmAbs (black circles=non-reactive; coloured circles=reactive) and control rmAbs (open circles) to citrullinated histone H2A peptides tested by ELISA (H2A1-21Cit; H2A27-47Cit; H2A69-90Cit; H2A79-98Cit; H2A94-113Cit) is shown. Results are expressed as absorbance at 405 nm. Each coloured circle represents an individual RA-rmAb.

We sorted single CD19+B cells from synovial cell suspension obtained from three joint replacements of patients with ELS+/ACPA+RA (figure 1A, B). Sequence analysis of different V_H_/J_H_ regions (n=139) and V_L_ regions (Vk=94;Vl=81) demonstrated that the V_H_/V_L_ gene repertoire of the synovial B cells was similar to PB CD5-IgM+B cells of healthy donors^9^ ^25^ (figure 1E–G). We observed statistically different JH gene usage with JH2/JH3 and JH5 over-represented in the IgM and IgG synovial B cells, respectively, and JH4 less frequent in the IgG compartment (figure 1E). Generally, we observed a similar frequency in the distribution of IgM (33%), IgG (40%) and IgA (27%) isotypes across all sequences analysed (figure 1C, D). IgG and IgA synovial B-cell clones showed significantly higher number of SHM in their V_H_ region compared with IgM, ∼50% of which displayed germline sequences (see figure 2A and online supplementary figure S1A); additionally, SHM in Variable Light (V_L_) was higher in κ than λ chains (figure 2B). Switched B-cell clones also showed (1) high ratios of replacement (R) to silent (S) mutations in CDR1-2 compared with the FR1-3 regions (see figure 2C and online supplementary figure S1B), (2) a shorter CDR3 length compared with unswitched unmutated IgM+clones (figure 2D) and (3) a higher frequency of positively charged aa, which have been shown to be frequently used by autoreactive B cells^26^ (figure 2E). We estimated that between 24% and 30% of the sequences (probability 0.05 and 0.1, respectively) from the synovial B cells display evidence of antigen selection (see online supplementary figure S2). Full antibodies characteristics are provided in online supplementary table S4. Clonal relationship analysis of the V_H_ gene sequences of RA synovial B cells showed evidence of intra-synovial diversification, as previously reported^9^ (figure 2f).

### RA synovial rmAbs display frequent immunoreactivity towards citrullinated histones

To investigate the autoantigenic immunoreactivity of the synovial B-cell clones, we cloned matching V_H_+V_L_ Ig genes from individual B cells into specific expression vectors and produced 66 in vitro whole rmAbs as complete IgG1 displaying identical V_H_+V_L_ pairing and specificity of the parental B cells.^21^ ^26^ Sufficient yield (>5 μg/mL) was obtained from 59 rmAbs (RA015/11=12; RA056/11=26; RA057/11=21), which were used for downstream analysis. First, we screened the RA-rmAbs in a synovial autoantigen microarray platform^27^ and in a multiplex RA-associated citrullinated antigen assay.^5^ Strikingly, RA-rmAbs (∼40%) showed strong immunoreactivity towards citrullinated histones H2A (citH2A) and citH2B by multiplex assay (figure 3A) with reactivity to histones H2A and H2B also frequently observed in the protein array heatmap (see online supplementary figure S3). Quantitative analysis confirmed that the strongest reactivity was directed against citH2A and citH2B followed by citrullinated vimentin and fibrinogen (figure 3B). Additionally, 5 rmAbs displayed binding to different citrullinated antigens, highlighting the existence of clones with multiple citrullinated reactivity. In particular, reactivity to citrullinated vimentin was demonstrated by ELISA in three RA-rmAbs (see online supplementary figure S4).

Overall, 41% (24/59) and 34% (20/59) of the RA-rmAbs were above the threshold of citH2A and citH2B reactivity, respectively (figure 3C). Such reactivity was confirmed to be disease-specific as it was not detectable in 30 control rmAbs from circulating naïve and memory B-cells of five patients with ANA+/ENA+ SS (see online supplementary figure S5). To confirm the importance of citrullination, we tested the RA-rmAbs towards the native versus citrullinated form of H2A and H2B by ELISA. A significant increase was detected in the binding to citH2A/H2B compared with native H2A/H2B histones in a large proportion of rmAbs from ELS+ACPA+RA synovial B cells but not in either naïve or memory SS B cells or flu control rmAb (figure 3D, E). Interestingly, using as substrate synthetic citrullinated H2A peptides spanning the whole histone H2A length, we showed that different antibodies each recognised different citrullinated H2A epitopes (figure 3F), suggesting the occurrence of in situ ‘epitope spreading’. The immunoreactivity observed against citrullinated histones or multiple citrullinated antigens was not due to polyreactivity, a phenomenon frequently observed in rmAbs generated from naïve B cells^28^ as only 1/59 clones displayed polyreactivity against multiple structurally unrelated antigens^21^ ^26^ (see online supplementary figure S6).

### RA synovial rmAbs with cit-histones specificity bind neutrophils NETs

**Figure 4:**
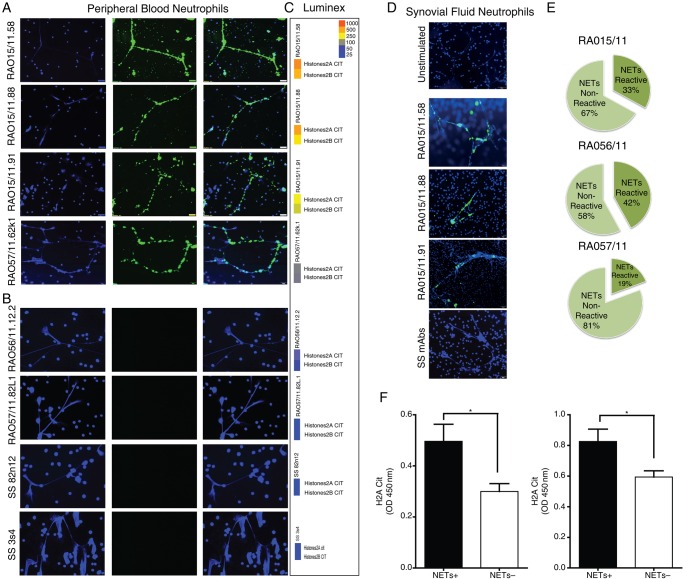
Rheumatoid arthritis (RA) synovial recombinant monoclonal antibodies (rmAbs) display selective immunoreactivity towards neutrophil extracellular traps (NETs). Representative pictures of PMA-stimulated neutrophils incubated with RA synovial (A) versus control Sjögren's syndrome (SS) rmAbs (B), demonstrating selective immunoreactivity of RA-rmAbs towards NETs. NETs are clearly evident as web-like structures rich in nuclear material stained by DAPI (blue, left columns) and are strongly bound by RA synovial (but not SS) rmAbs (green, middle columns, with overlap staining in the right columns). Corresponding multiplex tiles reporting the binding of the same rmAb towards citH2A and citH2B histones are reported beside each ImmunoFluorescence (IF) staining (C), demonstrating good accordance with anti-NET staining. (D) Binding of the RA synovial rmAbs to NETs is confirmed also in using synovial fluid neutrophils. Top image: RA SF-neutrophils seeded untreated onto cell culture cover slides for 30 min before fixation with 4% ParaFormAldehyde (PFA). A representative picture is shown. (E) Pie chart displaying the percentage of synovial rmAbs reacting towards NETs within individual synovial tissue demonstrated that up to 42% of the intra-synovial humoral response is directed towards NETs. (F) Subanalysis of the ELISA immunoreactivity towards citH2A and citH2B histones demonstrates significantly higher binding in anti-NET+ versus anti-NET− clones.

We next investigated the biological significant of the observed immunoreactivity towards citrullinated histones. First, we showed that the RA SF-neutrophils spontaneously undergo NETosis, as previously described^19^ (figure 4D, top). We assessed whether synovial RA-rmAbs reactive against citrullinated histones could specifically target NETs. A large proportion of RA-rmAbs displayed strong binding to NETs generated from either PB-neutrophils of healthy donors (figure 4A) or from RA SF-neutrophils (figure 4D) with 33%, 42% and 19% of the total synovial antibody response of patients RA015/11, RA056/11 and RA057/11, directed against NETs, respectively (figure 4E). Conversely, none of the control SS-rmAbs displayed anti-NET reactivity (figure 4B–D). Of relevance, the immunoreactivity of the RA-rmAbs was restricted to neutrophils undergoing NETosis with negligible or no binding to the nucleus of resting neutrophils.

Of relevance, the reactivity towards NETs was strongly associated with the level of immunobinding to citrullinated histones in the multiplex assay (see multiplex tiles in figure 4C) and in ELISA (figure 4F).

### Synovial B cells anti-NET immunoreactivity is dependent on SHM and is lost after reversion to germline

**Figure 5:**
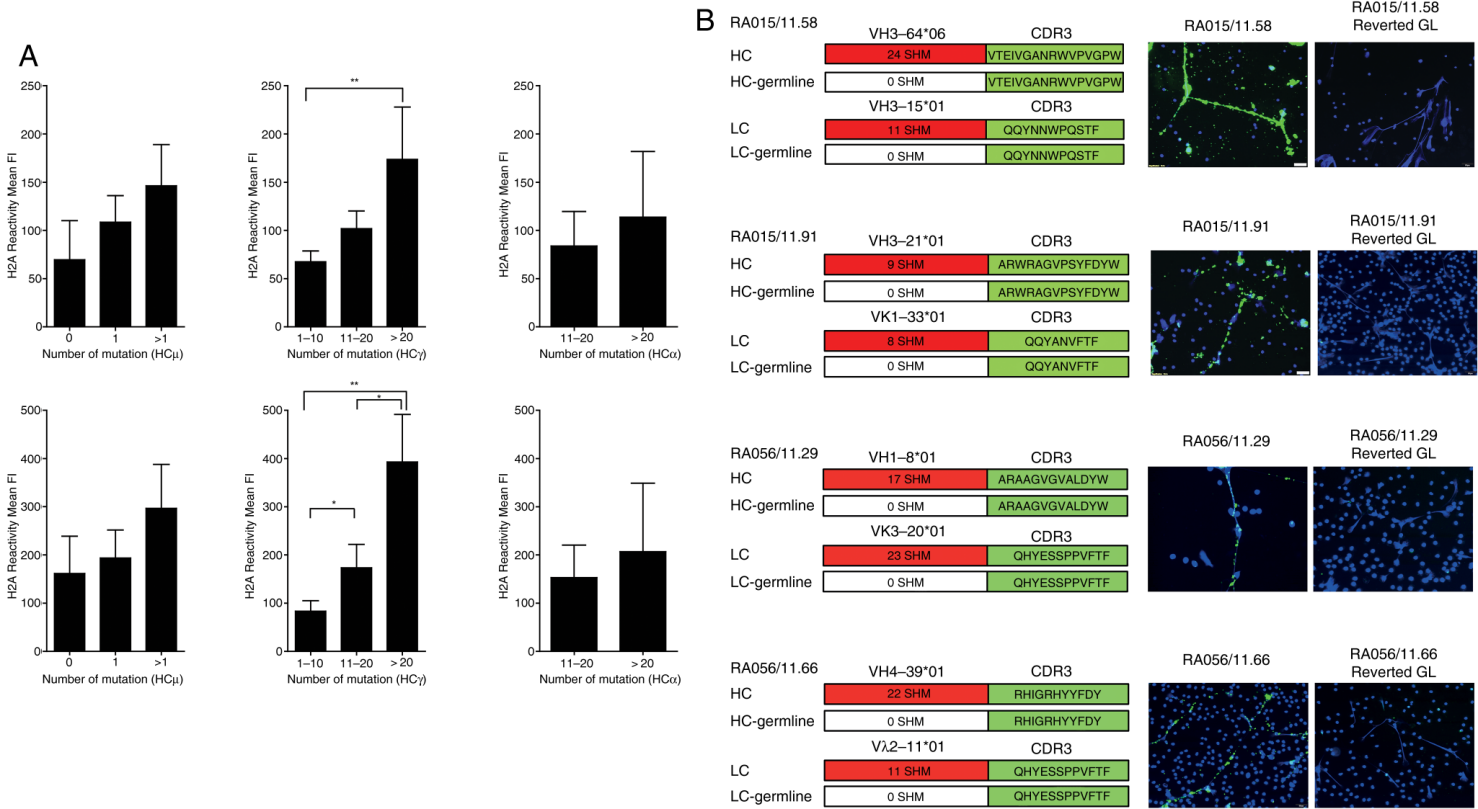
Immunoreactivity towards neutrophil extracellular traps (NETs) is dependent on somatic hypermutation. (A) Subanalysis of the anti-citH2A (top) and citH2B (bottom) histone reactivity in ELISA according to the number of somatic mutations in the VH regions of IgM (left), IgG (central) and IgA (right) clones demonstrates progressive increase in immunoreactivity according to the mutational load in all isotypes. (B) Reversal to germline (GL) sequences by overlapping PCR in representative individual anti-NET+ rheumatoid arthritis (RA) recombinant monoclonal antibody (rmAb) invariably abrogated the binding to NETs. The family usage, CDR3 sequence and the total number of somatic mutations in the FR and CDR regions of VH and VL Ig genes prior to reversal to GL sequences is shown beside each IF staining; *p<0.05; **p<0.01.

A progressive increase in the mutational load within the V_H_ Ig genes was associated with higher reactivity to citrullinated histones in all the isotypes obtained, with the strongest difference observed in IgG-switched B-cell clones (figure 5A). Therefore, to address the importance of affinity maturation and clonal diversification via SHM in the anti-NET reactivity, we reverted selected highly mutated RA-rmAbs with strong NETs reactivity to the corresponding V_H_+V_L_ Ig germline sequences by overlapping PCR. The germline RA-rmAbs invariably lost their reactivity towards NETs (figure 5B). These data demonstrate that intra-synovial antigen-driven SHM is required for the immunoreactivity of RA synovial B-cell clones to NET-associated autoantigens.

### ELS+RA synovia sustain anti-NET and anti-citrullinated histone antibodies in vivo in the Hu-RA/SCID chimeric mouse model

**Figure 6:**
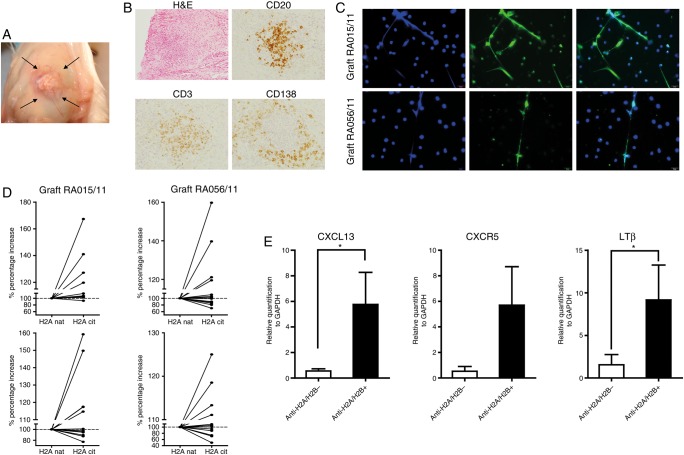
Ectopic lymphoid structures (ELS)+ rheumatoid arthritis (RA) synovia are self-maintained and release anti-neutrophil extracellular traps (NETs) and anti-citrullinated histone antibodies in vivo when engrafted in the Hu-RA/SCID chimeric model. (a) RA ELS+ synovial tissues RA015/11 and RA056/11 transplanted into SCID mice (arrow depict site of transplant) displayed persistent ELS after 4 weeks post-engraftment as shown in representative pictures of sequential analysis of paraffin-embedded sections stained for H&E and for CD20 (B cells), CD3 (T cells) and CD138 (plasma cells) (B). (C) Serum from Hu-RA SCID mice engrafted with RA015/11 and RA056/11 synovia reproduced the reactivity towards NETs in PMA-stimulated neutrophils. Representative pictures with NETs visualised by DAPI (blue) and the binding of human IgG in green are shown. (D) Binding of the human IgG in Hu-RA SCID mice to citrullinated versus unmodified H2A and H2B histones by ELISA confirmed the immunoreactivity observed with the recombinant monoclonal antibodies from the same patients. Results are shown as increased percentage in immunoreactivity in citrullinated versus native H2A and H2B histones. (E) Stratification of synovial RA grafts based on citH2A and citH2B immunoreactivity versus non-reactive demonstrated increased synovial expression of mRNA transcripts for CXCL13 and LTβ in anti-citH2A and citH2B reactive versus non-reactive samples; *p<0.05.

Finally, we investigated whether the anti-NET and anti-citH2A/H2B reactivity of the RA-rmAbs were reproduced in whole RA synovial tissue obtained from the same joints. For this purpose we used an in vivo chimeric human RA/SCID mouse transplantation model (figure 6A). As we had previously shown,^10^ RA synovial ELS were self-maintained for several weeks in the absence of recirculating immune cells (figure 6B) and released IgG-ACPA autoantibodies (measured as total anti-CCP-IgG, not shown). Mouse sera from mice transplanted with RA015/11 or RA056/11 synovial grafts contained autoreactive human anti-NET IgG (figure 6C) and/or anti-citH2A/citH2B histones antibodies (figure 6D). Additionally, in the synovial grafts of mice producing anti-citrullinated histones/NETs we observed significantly higher levels of CXCL13, CXCR5 and lymphotoxin (LT)β mRNA, which are master regulators of ectopic lymphoid neogenesis^29^ and are selectively upregulated in ELS+RA synovium^10^ ^30^ (figure 6E).

## Discussion

A common feature of RA is the formation of discrete clusters of infiltrating lymphomononuclear cells forming lymphoid aggregates or ELS.^7^ ^8^ ^10^ These structures resembling secondary lymphoid organs sustain a functional ectopic-GC response and support B-cell differentiation into high affinity autoantibody-producing cells.^29^ ^31^ ELS formation is dependent on the activation of the LT-β/lymphoid chemokines pathway in ectopic sites^32^ and is driven by chronic antigenic stimulation.^31^ As such, ELS (also observed in non-autoimmune conditions such as chronic infections, allograft rejections and cancer^31^) are unique in their ability to mount disease-specific and antigen-specific immune responses. Indeed, ectopic-GCs produce antibodies against citrullinated proteins in RA,^10^ ^33^ ^34^ ribonucleoproteins Ro/La in SS,^35^ ^36^ thyroglobulin and thyroperoxidase in Hashimoto's thyroiditis^37^ and acetylcholine receptor in myasthenia gravis.^38^

Here, we exploited these unique features of ectopic-GC to unravel the nature and source of the citrullinated antigens driving adaptive immune responses in the RA joints generating full rmAbs from the joints of patients with ACPA+RA containing functional ectopic-GCs. As control, due to the absence of a direct comparator as ELS synovitis do not harbour B cells for cloning,^10^ we used rmAbs generated from circulating naïve and Ag-experienced memory B cells of patients with SS to exclude that any observed reactivity was due to non-specific autoimmunity, as circulating SS B cells are frequently autoreactive.^22^

The rmAbs generated in this study have identical specificity of the parental B cells providing us the advantage of combining Ig gene repertoire and B-cell clonal analysis together with the characterisation of humoral immunoreactivity at clonal level.^26^ We adopted an unbiased approach sorting single CD19+ synovial tissue cells to have a true representation of the frequency and specificity of the autoimmune response against citrullinated antigens. Previous work generated rmAbs from RA synovial fluid IgG+ B cells and showed frequent reactivity to selected citrullinated antigens.^16^ Of relevance, synovial fluid rmAbs are not clonally related; conversely, the B-cell clones that we isolated displayed highly somatically hypermutated IgV_H_+V_L_ genes with evidence of intra-synovial clonal diversification compatible with their antigen-driven selection in ectopic-GC, similar to previous studies which microdissected ectopic-GC.^9^ This was particularly evident for IgG+ and IgA+ clones, while around 50% of the IgM+ B cells displayed germline sequences. As recently reported,^39^ we observed a high prevalence of IgA+ B cells in the RA joints in line with previous evidence that FDC-like synovial stromal cells directly induce IgA class-switch via a B cell Activating Factor of the TNF Family (BAFF) and A PRoliferation Inducing Ligand (APRIL)-dependent pathway.^40^ ^41^

Importantly, we demonstrated that around 40% of RA synovial B cells from ectopic-GCs displayed reactivity against citrullinated antigens. Although, we observed frequent cross-citrullinated reactivity, using a multiplex ‘citrullinome’ assay, we showed that the strongest reactivity of ACPA-producing B cells was directed towards citrullinated histones, mainly H2A and H2B, followed by cit-vimentin and cit-fibrinogen. The data on histone reactivity were further confirmed (1) in vitro using several immunoenzymatic methods and cell-based NETs co-localisation experiments and (2) in vivo by engrafting ELS+ synovial tissues into SCID mice where we showed that the synovial production of anti-citH2A/citH2B and anti-NET autoantibodies is critically dependent on ELS formation and is sustained by high levels of CXCL13 and LTβ. Although our work clearly suggest that cit-histones are critically targeted by synovial-derived ACPA, further work dissecting the fine specificities of the rmAbs towards multiple citrullinated antigens is warranted, also considering that the binding of ACPA can be greatly influenced by immunodominant epitope density and citrullination protocols in different assays.

Histone citrullination is a critical step in the formation of NETs released from neutrophils in response to infectious or inflammatory stimuli to trap and kill pathogens.^19^ Approximately 70% of all NETs proteins are histones in which arginine residues are deiminated to citrulline. Thus, NETosis allows the extracellular exposure of heavily citrullinated histones and, to a much lesser degree, also of other putative RA autoantigens such as citrullinated vimentin^19^ which can become the target of the autoimmune ACPA response. Interestingly, the reactivity towards citrullinated vimentin, which was relatively low in our antibodies, was recently described in work using rmAbs derived from synovial fluid B cells, which however did not investigate the reactivity towards citrullinated histones and NETs.^16^

The RA-rmAbs reactive towards citH2A/H2B also strongly and specifically recognised NETs in cell-based immunoassays using either RA-synovial fluid or circulating activated neutrophils as substrate. These data are highly relevant to RA pathophysiology since recent observations showed that (1) RA-synovial fluid neutrophils undergo aberrant NETosis and release citrullinated antigens^19^ and (2) patients with ACPA+RA sera recognise citrullinated histones from NETs.^2^

Importantly, the RA-rmAbs anti-NET immunoreactivity was entirely dependent on SHM in the IgV_H_ genes since it was completely abrogated when the IgV_H_+V_L_ genes were reverted into germline sequences. These data demonstrate that the anti-cit-histones/NET immunoreactivity is largely acquired within the synovial microenvironment on affinity maturation and intra-synovial diversification within ectopic-GC. Because these processes in B cells are largely dependent on cognate T-cell help, it is conceivable that Th cells with TCRs specific for processed NET peptides may be present in the RA synovium and engaged by antigen-presenting cells loading citrullinated NETs antigens. In this regard, recent evidence suggests that some NET proteins (eg, LL37, HMGB1) promote the activation of professional antigen-presenting cells by facilitating antigen uptake, interaction with endosomal Toll Like Receptor (TLR) and the release of type-I Interferon (IFN) and other proinflammatory cytokines as in the case of plasmacytoid DCs in SLE.^42^ ^43^ Similarly, in autoimmune vasculitis, NETs are highly immunogenic and mediate the transfer of cytoplasmic neutrophil antigens to myeloid dendritic cells, favouring the formation of anti-neutrophil cytoplasmic antibodies.^44^

The clearance of NETs by macrophages is an immunologically silent process. However, in an inflammatory environment, the uptake of NETs can promote proinflammatory cytokines including interleukin (IL)-1β, IL-6, and TNF-α,^45^ which are critical in RA. Their processing followed by presentation of deiminated antigens could elicit an adaptive immune response against citrullinated histones within GC in the RA joints, as also supported by long-standing evidence that synovial RA macrophages actively engulf neutrophil nuclear fragments.^46^ Although our and previous work suggests that NETosis is an important source of citrullinated proteins in the joints, several alternative/complementary mechanisms also likely contribute to the generation of citrullinated antigens and ACPA in RA both within and outside the joints. Environmental factors such as bacterial infection in periodontal disease (ie, *Porphyromonas gingivalis*)^47^ ^48^ and smoking^49^ can lead to the formation of citrullinated antigens at mucosal sites which, in susceptible individuals, can lead to the production of ACPA prior to the clinical onset.^4^ Within the RA joints, the activation of PADs by macrophages in the RA synovium can bring to the citrullination of intracellular and extracellular proteins such as vimentin and fibrin.^11–13^ Based on our data, we propose that NETosis is an additional critical source of citrullinated antigens within the synovium, whereby normally sequestered citrullinated antigens within neutrophils, such as core histones, can be hypercitrullinated, exposed and be targeted by the immune system within the RA joints. Although a formal mechanistic demonstration is still necessary, we propose that the local release of citrullinated histones contribute to fuel the antigen-driven generation of highly mutated B-cell clones within synovial ELS resulting in the generation of high-affinity ACPA displaying anti-NET reactivity (see online supplementary figure S8).

A critical question which remains to be elucidated is whether anti-NET antibodies generated in RA ELS are pathogenic and contribute to chronic inflammation over and above ACPA generated in the peripheral compartment, as the pathogenic role of ACPA remains controversial.^50^ ^51^ Additionally, it is unclear whether anti-NETs are generated in patients with early RA which display a similar prevalence of ELS+ synovitis compared with established RA.^52^ ^53^ The combination of next-generation sequencing followed by single cell cloning and recombinant antibody production together with the availability of ultrasound-guided synovial biopsies could help in clarifying this aspect.^52^ ^53^

## Supplementary Material

Web supplement

## References

[1] Schellekens GA, de Jong BA, van den Hoogen FH, et al Citrulline is an essential constituent of antigenic determinants recognized by rheumatoid arthritis-specific autoantibodies. J Clin Invest 1998;101:273–81. 10.1172/JCI1316 9421490 PMC508564

[2] Pratesi F, Dioni I, Tommasi C, et al Antibodies from patients with rheumatoid arthritis target citrullinated histone 4 contained in neutrophils extracellular traps. Ann Rheum Dis 2014;73:1414–22. 10.1136/annrheumdis-2012-202765 23727635

[3] Klareskog L, Amara K, Malmstrom V Adaptive immunity in rheumatoid arthritis: anticitrulline and other antibodies in the pathogenesis of rheumatoid arthritis. Curr Opin Rheumatol 2014;26:72–9. 10.1097/BOR.0000000000000016 24257366

[4] Rantapää-Dahlqvist S, de Jong BA, Berglin E, et al Antibodies against cyclic citrullinated peptide and IgA rheumatoid factor predict the development of rheumatoid arthritis. Arthritis Rheum 2003;48:2741–9. 10.1002/art.11223 14558078

[5] Sokolove J, Bromberg R, Deane KD, et al Autoantibody epitope spreading in the pre-clinical phase predicts progression to rheumatoid arthritis. PLoS ONE 2012;7:e35296 10.1371/journal.pone.0035296 22662108 PMC3360701

[6] Nielen MM, van Schaardenburg D, Reesink HW, et al Specific autoantibodies precede the symptoms of rheumatoid arthritis: a study of serial measurements in blood donors. Arthritis Rheum 2004;50:380–6. 10.1002/art.20018 14872479

[7] Takemura S, Braun A, Crowson C, et al Lymphoid neogenesis in rheumatoid synovitis. J Immunol 2001;167:1072–80. 10.4049/jimmunol.167.2.1072 11441118

[8] Manzo A, Bombardieri M, Humby F, et al Secondary and ectopic lymphoid tissue responses in rheumatoid arthritis: from inflammation to autoimmunity and tissue damage/remodeling. Immunol Rev 2010;233:267–85. 10.1111/j.0105-2896.2009.00861.x 20193005

[9] Scheel T, Gursche A, Zacher J, et al V-region gene analysis of locally defined synovial B and plasma cells reveals selected B cell expansion and accumulation of plasma cell clones in rheumatoid arthritis. Arthritis Rheum 2011;63:63–72. 10.1002/art.27767 20882667

[10] Humby F, Bombardieri M, Manzo A, et al Ectopic lymphoid structures support ongoing production of class-switched autoantibodies in rheumatoid synovium. PLoS Med 2009;6:e1 10.1371/journal.pmed.0060001 PMC2621263 19143467

[11] Vossenaar ER, Radstake TR, van der Heijden A, et al Expression and activity of citrullinating peptidylarginine deiminase enzymes in monocytes and macrophages. Ann Rheum Dis 2004;63:373–81. 10.1136/ard.2003.012211 15020330 PMC1754951

[12] Foulquier C, Sebbag M, Clavel C, et al Peptidyl arginine deiminase type 2 (PAD-2) and PAD-4 but not PAD-1, PAD-3, and PAD-6 are expressed in rheumatoid arthritis synovium in close association with tissue inflammation. Arthritis Rheum 2007;56:3541–53. 10.1002/art.22983 17968929

[13] Masson-Bessiere C, Sebbag M, Girbal-Neuhauser E, et al The major synovial targets of the rheumatoid arthritis-specific antifilaggrin autoantibodies are deiminated forms of the alpha- and beta-chains of fibrin. J Immunol 2001;166:4177–84. 10.4049/jimmunol.166.6.4177 11238669

[14] Kinloch A, Tatzer V, Wait R, et al Identification of citrullinated alpha-enolase as a candidate autoantigen in rheumatoid arthritis. Arthritis Res Ther 2005;7:R1421–9. 10.1186/ar1845 16277695 PMC1297593

[15] De Rycke L, Nicholas AP, Cantaert T, et al Synovial intracellular citrullinated proteins colocalizing with peptidyl arginine deiminase as pathophysiologically relevant antigenic determinants of rheumatoid arthritis-specific humoral autoimmunity. Arthritis Rheum 2005;52:2323–30. 10.1002/art.21220 16052592

[16] Amara K, Steen J, Murray F, et al Monoclonal IgG antibodies generated from joint-derived B cells of RA patients have a strong bias toward citrullinated autoantigen recognition. J Exp Med 2013;210:445–55. 10.1084/jem.20121486 23440041 PMC3600900

[17] Wang Y, Li M, Stadler S, et al Histone hypercitrullination mediates chromatin decondensation and neutrophil extracellular trap formation. J Cell Biol 2009;184:205–13. 10.1083/jcb.200806072 19153223 PMC2654299

[18] Urban CF, Ermert D, Schmid M, et al Neutrophil extracellular traps contain calprotectin, a cytosolic protein complex involved in host defense against Candida albicans. PLoS Pathog 2009;5:e1000639 10.1371/journal.ppat.1000639 19876394 PMC2763347

[19] Khandpur R, Carmona-Rivera C, Vivekanandan-Giri A, et al NETs are a source of citrullinated autoantigens and stimulate inflammatory responses in rheumatoid arthritis. Sci Transl Med 2013;5:178ra140 10.1126/scitranslmed.3005580 PMC3727661 23536012

[20] Aletaha D, Neogi T, Silman AJ III, et al 2010 Rheumatoid arthritis classification criteria: an American College of Rheumatology/European League Against Rheumatism collaborative initiative. Arthritis Rheum 2010;62:2569–81. 10.1002/art.27584 20872595

[21] Tiller T, Meffre E, Yurasov S, et al Efficient generation of monoclonal antibodies from single human B cells by single cell RT-PCR and expression vector cloning. J Immunol Methods 2008;329:112–24. 10.1016/j.jim.2007.09.017 17996249 PMC2243222

[22] Corsiero E, Sutcliffe N, Pitzalis C, et al Accumulation of self-reactive naive and memory B cell reveals sequential defects in B cell tolerance checkpoints in Sjogren's syndrome. PLoS ONE 2014;9:e114575 10.1371/journal.pone.0114575 25535746 PMC4275206

[23] Rogosch T, Kerzel S, Hoi KH, et al Immunoglobulin analysis tool: a novel tool for the analysis of human and mouse heavy and light chain transcripts. Front Immunol 2012;3:176 10.3389/fimmu.2012.00176 22754554 PMC3384897

[24] English D, Andersen BR Single-step separation of red blood cells. Granulocytes and mononuclear leukocytes on discontinuous density gradients of Ficoll-Hypaque. J Immunol Methods 1974;5:249–52. 10.1016/0022-1759(74)90109-4 4427075

[25] Brezinschek HP, Foster SJ, Brezinschek RI, et al Analysis of the human VH gene repertoire. Differential effects of selection and somatic hypermutation on human peripheral CD5(+)/IgM+ and CD5(-)/IgM+ B cells. J Clin Invest 1997;99: 2488–501. 10.1172/JCI119433 9153293 PMC508090

[26] Wardemann H, Yurasov S, Schaefer A, et al Predominant autoantibody production by early human B cell precursors. Science 2003;301:1374–7. 10.1126/science.1086907 12920303

[27] Robinson WH, DiGennaro C, Hueber W, et al Autoantigen microarrays for multiplex characterization of autoantibody responses. Nat Med 2002;8:295–301. 10.1038/nm0302-295 11875502

[28] Wardemann H, Nussenzweig MC B-cell self-tolerance in humans. Adv Immunol 2007;95:83–110. 10.1016/S0065-2776(07)95003-8 17869611

[29] Aloisi F, Pujol-Borrell R Lymphoid neogenesis in chronic inflammatory diseases. Nat Rev Immunol 2006;6:205–17. 10.1038/nri1786 16498451

[30] Weyand CM, Seyler TM, Goronzy JJ B cells in rheumatoid synovitis. Arthritis Res Ther 2005;7 Suppl 3:S9–12. 10.1186/ar1737 15960820 PMC2833971

[31] Pitzalis C, Jones GW, Bombardieri M, et al Ectopic lymphoid-like structures in infection, cancer and autoimmunity. Nat Rev Immunol 2014;14:447–62. 10.1038/nri3700 24948366

[32] Ansel KM, Ngo VN, Hyman PL, et al A chemokine-driven positive feedback loop organizes lymphoid follicles. Nature 2000;406:309–14. 10.1038/35018581 10917533

[33] Croia C, Serafini B, Bombardieri M, et al Epstein-Barr virus persistence and infection of autoreactive plasma cells in synovial lymphoid structures in rheumatoid arthritis. Ann Rheum Dis 2013;72:1559–68. 10.1136/annrheumdis-2012-202352 23268369

[34] Masson-Bessiere C, Sebbag M, Durieux JJ, et al In the rheumatoid pannus, anti-filaggrin autoantibodies are produced by local plasma cells and constitute a higher proportion of IgG than in synovial fluid and serum. Clin Exp Immunol 2000;119:544–52. 10.1046/j.1365-2249.2000.01171.x 10691929 PMC1905590

[35] Salomonsson S, Jonsson MV, Skarstein K, et al Cellular basis of ectopic germinal center formation and autoantibody production in the target organ of patients with Sjogren's syndrome. Arthritis Rheum 2003;48:3187–201. 10.1002/art.11311 14613282

[36] Croia C, Astorri E, Murray-Brown W, et al Implication of Epstein-Barr virus infection in disease-specific autoreactive B cell activation in ectopic lymphoid structures of Sjogren's syndrome. Arthritis Rheum 2014;66:2545–57. 10.1002/art.38726 24891330

[37] Armengol MP, Juan M, Lucas-Martín A, et al Thyroid autoimmune disease: demonstration of thyroid antigen-specific B cells and recombination-activating gene expression in chemokine-containing active intrathyroidal germinal centers. Am J Pathol 2001;159:861–73. 10.1016/S0002-9440(10)61762-2 11549579 PMC1850445

[38] Berrih-Aknin S, Ragheb S, Le Panse R, et al Ectopic germinal centers, BAFF and anti-B-cell therapy in myasthenia gravis. Autoimmun Rev 2013;12:885–93. 10.1016/j.autrev.2013.03.011 23537509

[39] Bos WH, van de Stadt LA, Sohrabian A, et al Development of anti-citrullinated protein antibody and rheumatoid factor isotypes prior to the onset of rheumatoid arthritis. Arthritis Res Ther 2014;16:405 10.1186/ar4511 25167340 PMC4060448

[40] Bombardieri M, Kam NW, Brentano F, et al A BAFF/APRIL-dependent TLR3-stimulated pathway enhances the capacity of rheumatoid synovial fibroblasts to induce AID expression and Ig class-switching in B cells. Ann Rheum Dis 2011;70:1857–65. 10.1136/ard.2011.150219 21798884

[41] Alsaleh G, Francois A, Knapp AM, et al Synovial fibroblasts promote immunoglobulin class switching by a mechanism involving BAFF. Eur J Immunol 2011;41:2113–22. 10.1002/eji.201041194 21557212

[42] Lande R, Gregorio J, Facchinetti V, et al Plasmacytoid dendritic cells sense self-DNA coupled with antimicrobial peptide. Nature 2007;449:564–9. 10.1038/nature06116 17873860

[43] Garcia-Romo GS, Caielli S, Vega B, et al Netting neutrophils are major inducers of type I IFN production in pediatric systemic lupus erythematosus. Sci Transl Med 2011;3:73ra20 10.1126/scitranslmed.3001201 PMC3143837 21389264

[44] Sangaletti S, Tripodo C, Chiodoni C, et al Neutrophil extracellular traps mediate transfer of cytoplasmic neutrophil antigens to myeloid dendritic cells toward ANCA induction and associated autoimmunity. Blood 2012;120: 3007–18. 10.1182/blood-2012-03-416156 22932797

[45] Farrera C, Fadeel B Macrophage clearance of neutrophil extracellular traps is a silent process. J Immunol 2013;191:2647–56. 10.4049/jimmunol.1300436 23904163

[46] Savill JS, Wyllie AH, Henson JE, et al Macrophage phagocytosis of aging neutrophils in inflammation. Programmed cell death in the neutrophil leads to its recognition by macrophages. J Clin Invest 1989;83:865–75. 10.1172/JCI113970 2921324 PMC303760

[47] Wegner N, Wait R, Sroka A, et al Peptidylarginine deiminase from Porphyromonas gingivalis citrullinates human fibrinogen and alpha-enolase: implications for autoimmunity in rheumatoid arthritis. Arthritis Rheum 2010;62:2662–72. 10.1002/art.27552 20506214 PMC2941529

[48] Lee JY, Choi IA, Kim JH, et al Association between anti-Porphyromonas gingivalis or anti-alpha-enolase antibody and severity of periodontitis or rheumatoid arthritis (RA) disease activity in RA. BMC Musculoskelet Disord 2015;16:190 10.1186/s12891-015-0647-6 26265263 PMC4542108

[49] Kokkonen H, Brink M, Hansson M, et al Associations of antibodies against citrullinated peptides with human leukocyte antigen-shared epitope and smoking prior to the development of rheumatoid arthritis. Arthritis Res Ther 2015;17:125 10.1186/s13075-015-0638-x 25990747 PMC4438519

[50] Kuhn KA, Kulik L, Tomooka B, et al Antibodies against citrullinated proteins enhance tissue injury in experimental autoimmune arthritis. J Clin Invest 2006;116:961–73. 10.1172/JCI25422 16585962 PMC1421345

[51] Chirivi RGS, Jenniskens GJ, Raats JMH Anti-citrullinated protein antibodies as novel therapeutic drugs in rheumatoid arthritis. Clin Cell Immunol 2013;S6 doi:10.4172/2155-9899.S6-006 10.4172/2155-9899.S6-006

[52] Kelly S, Humby F, Filer A, et al Ultrasound-guided synovial biopsy: a safe, well-tolerated and reliable technique for obtaining high-quality synovial tissue from both large and small joints in early arthritis patients. Ann Rheum Dis 2015;74:611–17. 10.1136/annrheumdis-2013-204603 24336336

[53] Pitzalis C, Kelly S, Humby F New learnings on the pathophysiology of RA from synovial biopsies. Curr Opin Rheumatol 2013;25:334–44. 10.1097/BOR.0b013e32835fd8eb 23492740

