# Supplementary material for: Single cell cloning and recombinant monoclonal antibodies generation from RA synovial B cells reveal frequent targeting of citrullinated histones of NETs
Source: Ann Rheum Dis. 2015 Dec 9;75(10):1866–75. doi: 10.1136/annrheumdis-2015-208356 (PMC5036240; doi:10.1136/annrheumdis-2015-208356)
Supplement: Web supplement [file annrheumdis-2015-208356-s1.pdf]

Supplementary Materials and Methods

Synovial mononuclear cell isolation and CD19+ cell FACS sorting

The synovial tissue was cut into small pieces and enzymatically digested with collagenase D (100 mg/ml) and DNase I (10 mg/ml) at 37 °C for 1 hour. 0.5 M EDTA was added to stop the reaction. The samples were then filtered through 40-μm cell strainer to remove undigested tissue. Cells viability was determined by Trypan blue exclusion test. For flow cytometry cells were stained with PerCP-Cy5.5-anti-human CD19 (clone-SJ25C1; BD Biosciences) and FITC-anti-human CD3 (clone-HIT3a; eBioscience). Flow cytometric analysis and sorting was performed with a FACSAria flow cytometer (Becton Dickinson). Single CD19+ cells were sorted directly into 96-well plates, as previously described [21].

Synovial tissue histological characterization of lymphocytic aggregates

Sequential paraffin-embedded 3μm sections of synovial tissue were stained for the markers CD3 (1:80 dilution; DAKO), CD20 (1:50 dilution; DAKO) and CD138 (1:50 dilution; DAKO) following routine H&E staining to classify the lymphocytic infiltration as aggregate or diffuse, as previously reported [10].

Synovial antigen microarray profiling

The synovial antigen microarray production, probing and scanning protocol has been previously described [27]. Briefly, arrays were probed with 10 μg/ml rmAbs for 1 hour at 4°C followed by washing and incubation with Cy3-conjugated goat anti-human secondary antibody. The arrays were scanned using a GenePix 4400A scanner and the net mean pixel intensities of each feature were determined using GenePix Pro 7.0 software. The net median pixel intensity of each feature above the background was used.

Arginine deimination of histone H2A and H2B

Histones H2A and H2B purified from bovine thymus tissue were incubated at 1 mg/ml with rabbit skeletal muscle PAD (7 U/mg) in 0.1 M Tris-HCl (pH 7.4), 10 mM $CaCl_2$, and 5 mM DTT for 2 h at 50°C. After incubation each histone was stored at -80 °C in aliquots of 100 µl each.

ELISA for anti-citH2A peptides

ELISA plates were coated with citrullinated or peptides derived from H2A at 10 µg/ml in PBS and incubated over-night at 4°C. Samples at 10 µg/ml diluted in PBS, 0.5% Porcine gelatin, 0.05% Tween-20 were transferred into the ELISA plate and incubated for 2 hours at RT. Unbound antibodies were removed by washings before incubation for 2 hour RT with Alkaline-Phosphatase-coupled goat anti-human IgG. OD were measured at 405 nm.

ELISA for anti-mutated citrullinated vimentin (MCV)

Samples were tested for reactivity against mutated citrullinated vimentin (MCV) using commercially available ELISA kit (Orgentec anti-MCV). The ELISA test was performed according to the manufacturer's instructions. Samples were added to the antigen coated plate and incubated 1 hour at RT at a concentration of 10 µg/ml. OD were measured at 450 nm.

Characterization of polyreactivity by ELISA

To test the reactivity against different allo- and auto-antigens, supernatants were tested for polyreactivity against double and single-stranded DNA (dsDNA and ssDNA), lipopolysaccharide (LPS) and insulin by ELISA as previously reported [21]. Antibodies that reacted against at least two structurally diverse self- and non-self-antigens were defined as polyreactive [21, 26]. Internal controls for polyreactivity were added on each plate consisting of the recombinant monoclonal antibodies mGO53 (negative), JB40 (low polyreactive), and ED38 (highly polyreactive) as previously reported [21].

Overlap-PCR to revert mutated IgH and IgL chain genes to germline sequence

Antibodies for reversion experiments are listed in **Table S3** and included 5 synovial rmAbs with a strong reactivity in fluorescence microscopy on NETs. Mutated VH+VL regions were reverted into their germline (GL) counterpart sequence using a previously described overlap strategy [21]. This consisted of two (if J gene germline) or three (if J gene mutated) independent first PCR reactions followed by a nested overlapping PCR to join the amplicons generated with the first PCRs. As templates for the first reactions we used plasmids containing the rmAbs clone specific CDR3 regions and plasmids derived from naïve B-cells, containing the corresponding unmutated VH and VL genes (**Fig.S7**). PCRs were performed at 98 °C(1 min), 98 °C(20 sec), 56 °C(20 sec), 72 °C(20 sec), and 72 °C(2 min) for 32 cycles using the Q5-High-Fidelity DNA polymerase (NEB). Reverted IgH+IgL chain PCR products were sequenced to confirm the absence of mutations. GL antibodies were expressed and tested in fluorescence microscopy on NETs as described above. For the full list of primers used in the overlap PCRs see **Table S3**.

RA synovial tissue transplantation into SCID mice

Human arthroplasty synovium from the same 2 RA patients (RA015/11 and RA056/11) from which the monoclonal antibodies were generated was transplanted subcutaneously into Beige SCID-17 mice as previously described [10]. A total of 31 SCID mice were transplanted with synovial tissues from either patients. Four weeks post-transplantation animals were sacrificed and underwent terminal bleed. Serum was collected and stored at -20°C for subsequent analysis of human APCA, anti-NET and anti-citrullinated histone antibodies. Furthermore, at culling each synovial graft was harvested and divided into two parts; one part was paraffin embedded for later histological characterization and one part was stored in RNA-later at -80°C for quantitative real-time RT-PCR. All procedures were performed according to the Home Office regulations (PPL 70/7001).

Supplementary Figures

Figure S1: Single synovial CD19+ VH/VL Ig gene analysis for each synovial tissue.

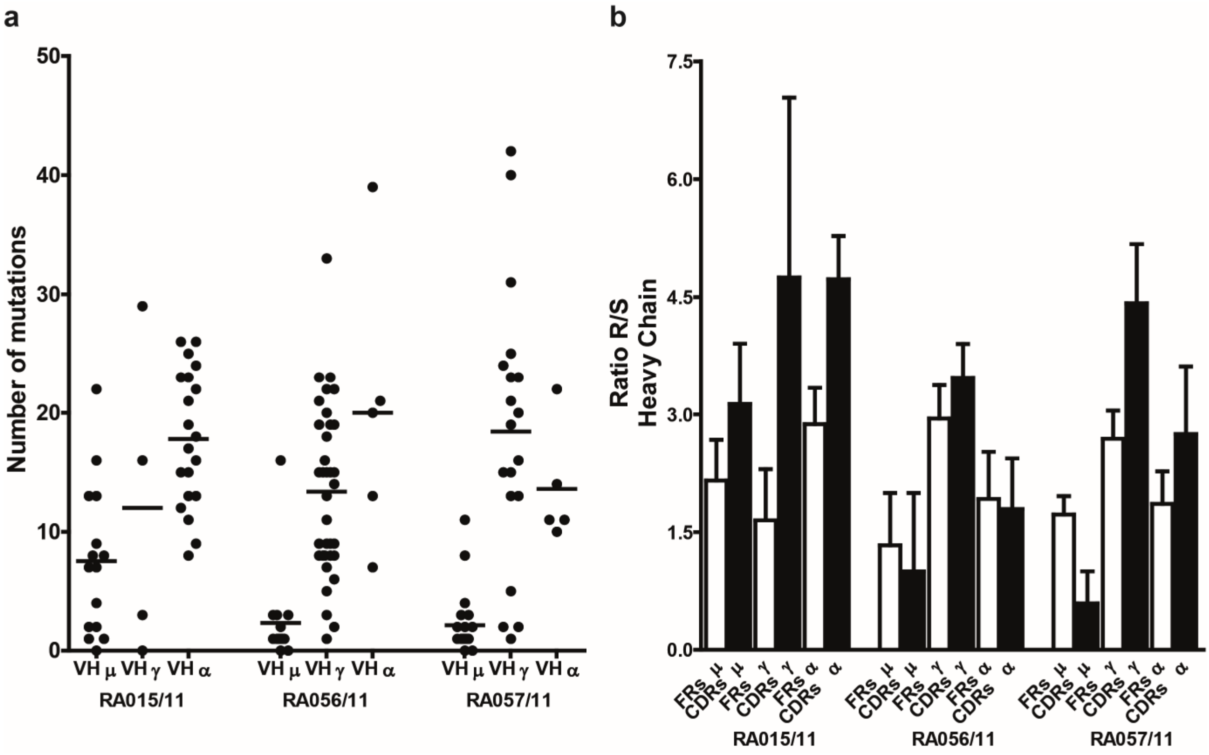

(a) Frequencies of μ, γ, and α heavy chain among all CD19+ B cells for each synovial tissue. **(b)** Absolute numbers of somatic mutations in VH genes for IgM, IgG and IgA for each synovial tissue. **(c)** Frequency of replacement (R) and silent (S) mutation ratio in FR (white) and CDR (black) regions for IgM, IgG and IgA for each synovial tissue.

Figure S2: Interference of antigen selection in RA-rmAbs.

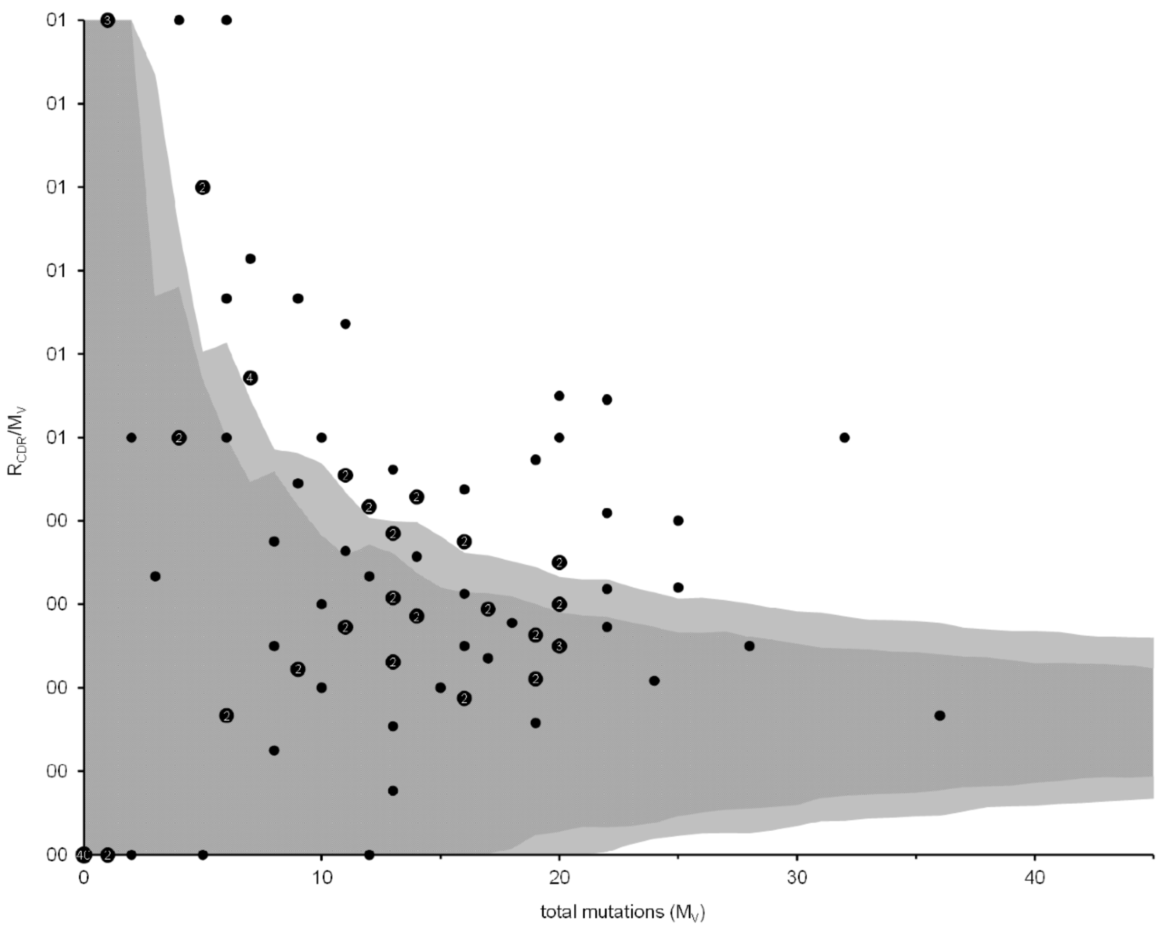

The graph shows the ratio of replacement mutations in CDR1 and CDR2 (R_{CDR}) to the total number of mutations in V region (M_V) plotted against M_V for the RA-rmAbs. The dark and the light grey area indicate the 90% and 95% confidence limits for the probability of random mutations, respectively. A data point outside these areas represents a sequence that was antigen selected. The data were obtained using the Immunoglobulin Analysis Tool software [22].

Figure S3: Overall heatmap illustrating the binding of all RA synovial rmAbs in the autoantigen array.

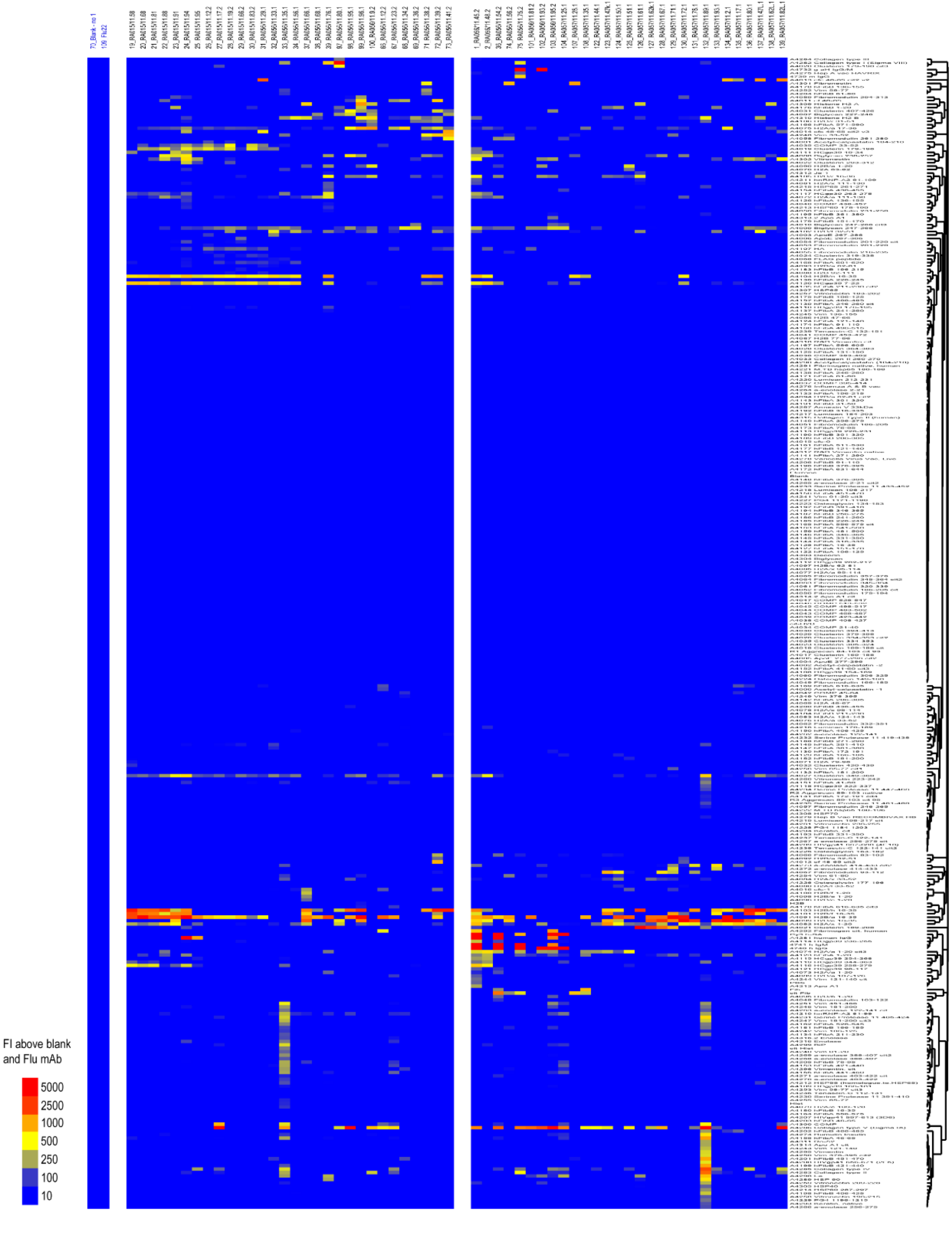

RA-rmAbs antigen array reactivity. Heatmap tiles reflect the amount of IgG autoantibody binding reactivity based on the fluorescence intensity scale as indicated on the left bottom. Samples and antigens are shown in columns and rows, respectively. Blank and control flu antibody are reported on the left side of the heatmap.

Figure S4: Immunoreactivity towards citrullinated vimentin.

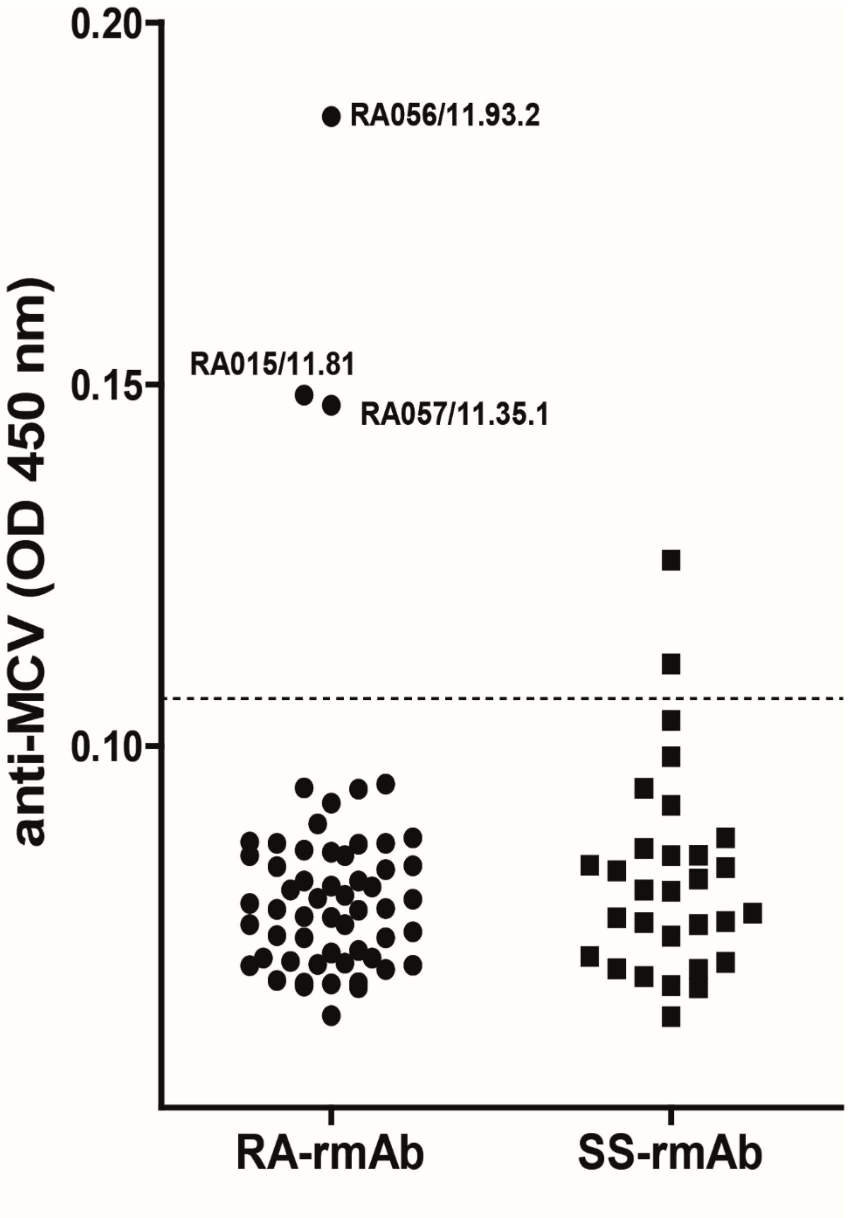

Binding of the RA and control rmAbs (30 naïve and memory B-cell clones from SS patients) to mutated citrullinated vimentin (MCV) tested by ELISA. The dotted horizontal line represents the cut-off for positivity of the rmAbs which was determined as the mean+2SD of the reactivity of 30 SS control rmAbs (right panel).

Figure S5: Multiplex citrullinated antigen assay.

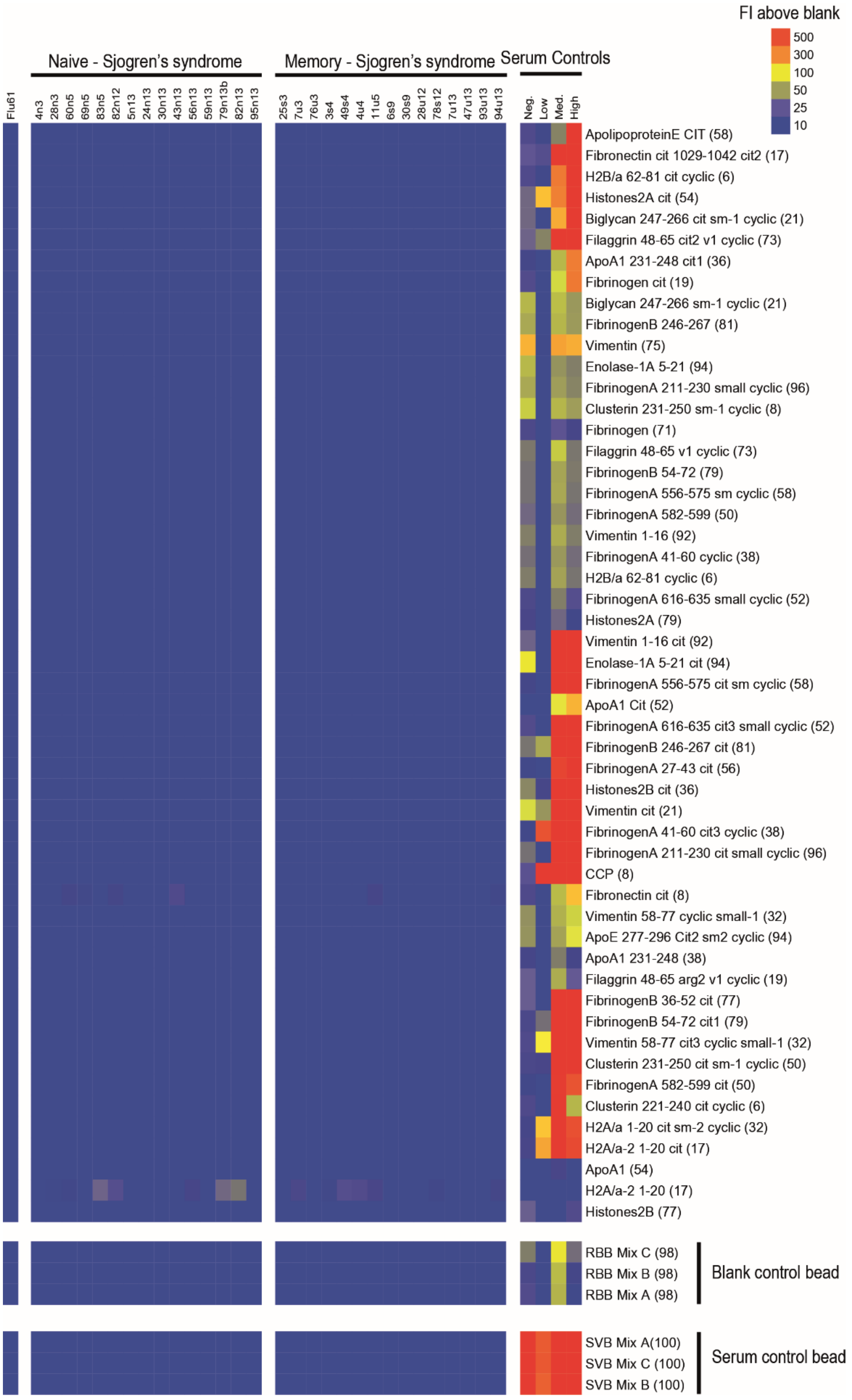

Luminex heatmap for control rmAbs derived from naïve and memory B-cells of SS patients. Heatmap tiles reflect the amount of IgG autoantibody binding reactivity based on the fluorescence intensity scale as indicated on the right top. Samples and synovial antigens are shown in columns and rows, respectively. ACPA negative and ACPA low, medium and high-reactive RA sera are included on the right hand side column. Blank and serum control beads are reported at the bottom of the heatmap.

Figure S6: Polyreactivity analysis of synovial rmAbs.

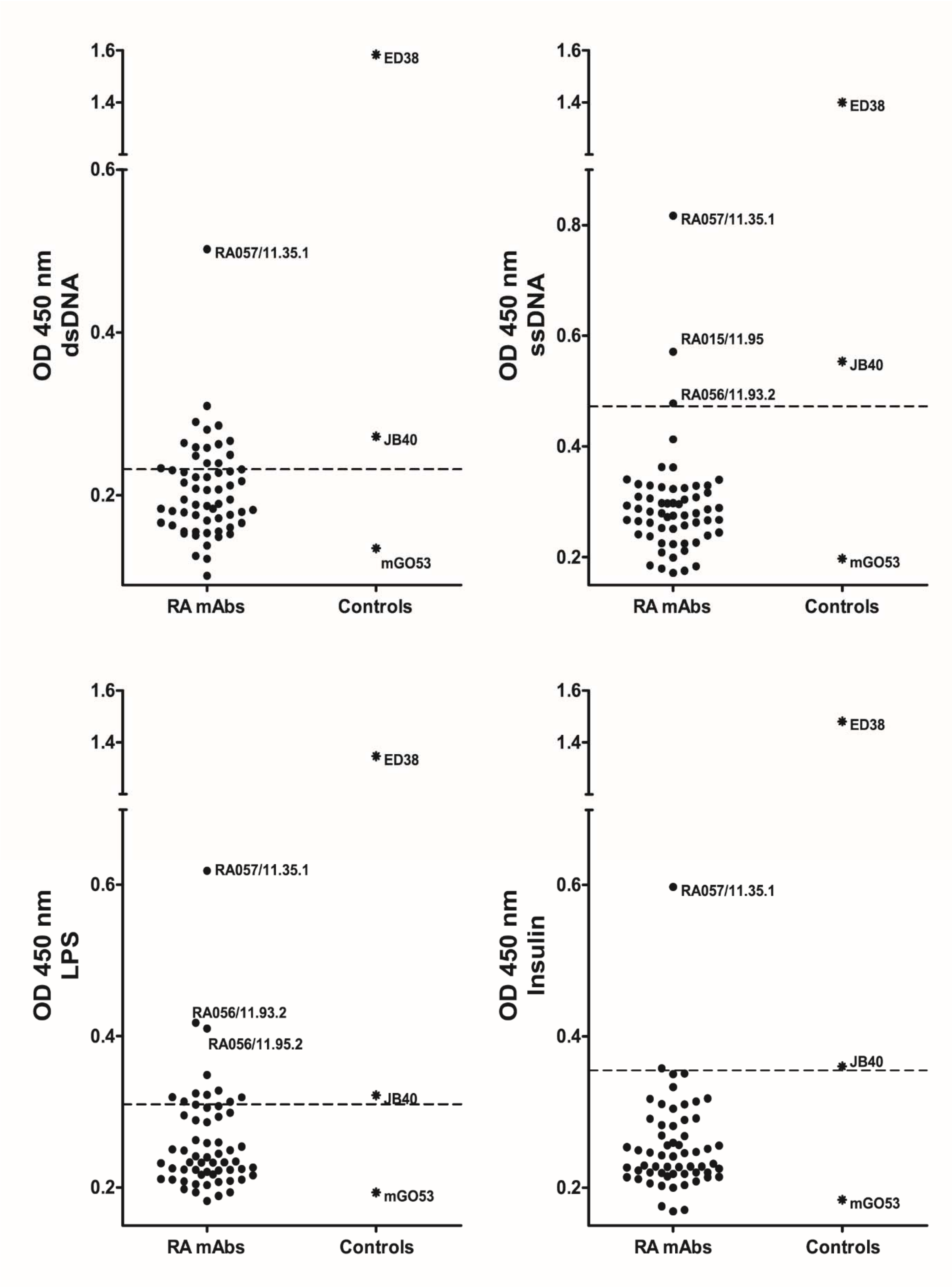

Synovial rmAbs were tested for reactivity with dsDNA (top left), ssDNA (top right), LPS (bottom left) and insulin (bottom right) by ELISA. Each graph shows the reactivity at a concentration of 1 μg/ml and it shows the result of two independent experiments. The cut-off OD (450 nm) at which antibodies were considered reactive is shown by the horizontal lines. Data points represent individual antibodies. Internal controls for polyreactivity are shown in each graph and include mGO53 (negative [26]), JB40 (low polyreactive [26]), and ED38 (highly polyreactive [26]).

Figure S7: Overlapping PCR strategy.

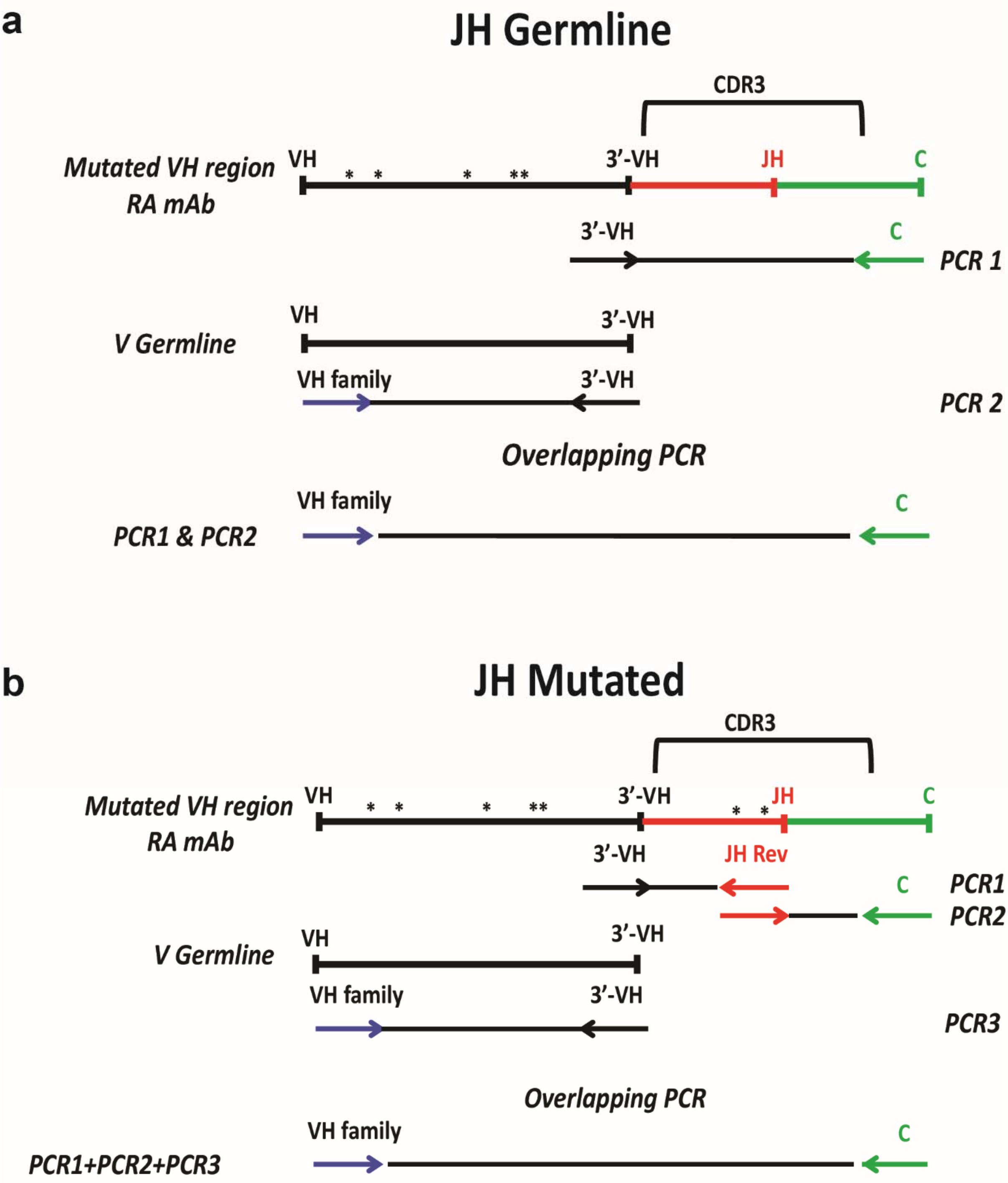

Strategy to revert mutated Ig genes into their unmutated counterpart by **(a)** two (J gene germline) or **(b)** three (J gene mutated) separate PCRs for the V gene and the (D)J gene, followed by a final overlapping PCR to join the two PCR products.

Figure S8: Neutrophil NETosis and anti-NET antibodies in synovial ELS: a new model suggesting linking inflammation and autoimmunity to citrullinated proteins in RA.

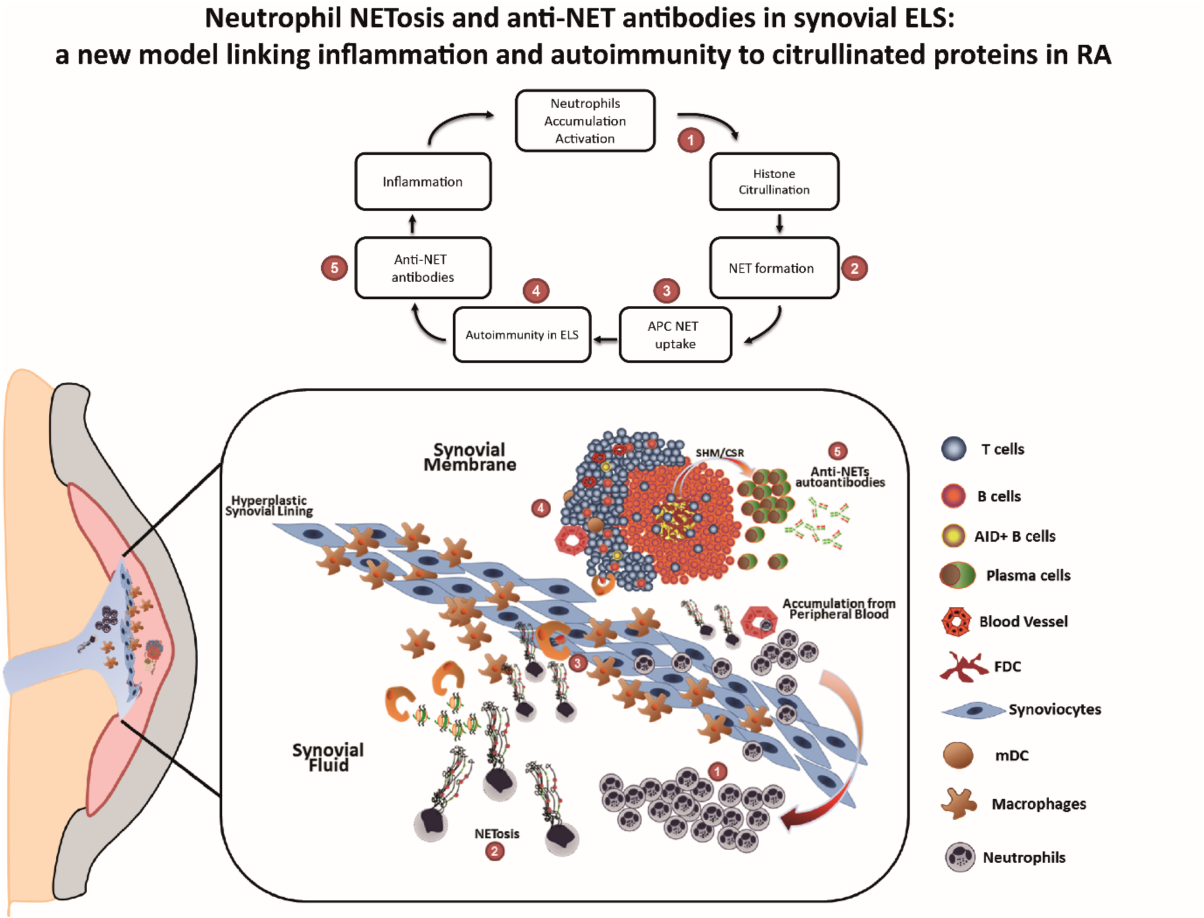

Migration of neutrophils from the periphery followed by aberrant neutrophil NETosis during chronic inflammation within the RA compartment (synovial tissue and fluid, steps 1-2) provide a continuous source of externalised citrullinated antigens, such as citH2A, citH2B and citH4 histones that can be presented by antigen-presenting cells following engulfment of neutrophils nuclear fragments (step 3). Such process would sustain an antigen-driven autoimmune response towards citrullinated antigens within ELS in the RA joint (step 4) resulting in the production of high affinity ACPA displaying anti-NET reactivity (step 5) which may contribute to the perpetuation of chronic inflammation and autoimmunity

Table S1. Complete list of primers used in this study

1st PCR HC	Forward 5' to 3'
5' L-VH 1	ACAGGTGCCCACTCCCAGGTGCAG
5' L-VH 3	AAGGTGTCCAGTGTGARGTGCAG
5' L-VH 4/6	CCCAGATGGGTCCTGTCCCAGGTGCAG
5' L-VH 5	CAAGGAGTCTGTTCCGAGGTGCAG
	Reverse 3' to 5'
3' Cμ CH1 ext	GGAAGGAAGTCCTGTGCGAGGC
3' Cγ CH1 ext	GGAAGGTGTGCACGCCGCTGGTC
3' Cα CH1 ext	TGGGAAGTTTCTGGCGGTCACG
2nd PCR HC	Forward 5' to 3'
5' AgeI VH1	CTGCAACCGGTGTACATTCCCAGGTGCAGCTGGTGCAG
5' AgeI VH1/5	CTGCAACCGGTGTACATTCCGAGGTGCAGCTGGTGCAG
5' AgeI VH 1-18	CTGCAACCGGTGTACATTCCCAGGTTCAGCTGGTGCAG
5' AgeI VH 1-24	CTGCAACCGGTGTACATTCCCAGGTCCAGCTGGTACAG
5' AgeI VH3	CTGCAACCGGTGTACATTCTGAGGTGCAGCTGGTGGAG
5' AgeI VH3-23	CTGCAACCGGTGTACATTCTGAGGTGCAGCTGTTGGAG
5' AgeI VH3-33	CTGCAACCGGTGTACATTCTCAGGTGCAGCTGGTGGAG
5' AgeI VH 3-9	CTGCAACCGGTGTACATTCTGAAGTGCAGCTGGTGGAG
5' AgeI VH4	CTGCAACCGGTGTACATTCCCAGGTGCAGCTGCAGGAG
5' AgeI VH 4-34	CTGCAACCGGTGTACATTCCCAGGTGCAGCTACAGCAGTG
5' AgeI VH4-39	CTGCAACCGGTGTACATTCCCAGCTGCAGCTGCAGGAG
5' AgeI VH 6-1	CTGCAACCGGTGTACATTCCCAGGTACAGCTGCAGCAG
	Reverse 3' to 5'
3' Cμ CH1 int	GGGAATTCTCACAGGAGACGA
3' Cγ IgG int	GTTCGGGGAAGTAGTCCTTGAC
3' Cα CH1-2 int	GTCCGCTTTCGCTCCAGGTCACACT
1st PCR λ LC	Forward 5' to 3'
5' L Vλ 1	GGTCCTGGGCCCAGTCTGTGCTG
5' L Vλ 2	GGTCCTGGGCCCAGTCTGCCCTG
5' L Vλ 3	GCTCTGTGACCTCCTATGAGCTG
5' L Vλ 4/5	GGTCTCTCTCSCAGCYTGTGCTG
5' L Vλ 6	GTTCTTGGGCCAATTTTATGCTG
5' L Vλ 7	GGTCCAATTCYCAGGCTGTGGTG
5' L Vλ 8	GAGTGGATTCTCAGACTGTGGTG
	Reverse 3' to 5'
3' Cλ	CACCAGTGTGGCCTTGTTGGCTTG
2nd PCR l LC	Forward 5' to 3'
5' AgeI Vλ 1	CTGCTACCGGTTCCTGGGCCCAGTCTGTGCTGACKCAG
5' AgeI Vλ 2	CTGCTACCGGTTCCTGGGCCCAGTCTGCCCTGACTCAG
5' AgeI Vλ 3	CTGCTACCGGTTCTGTGACCTCCTATGAGCTGACWCAG
5' AgeI Vλ 4/5	CTGCTACCGGTTCTCTCTCSCAGCYTGTGCTGACTCA
5' AgeI Vλ 6	CTGCTACCGGTTCTTGGGCCAATTTTATGCTGACTCAG
5' AgeI Vλ 7/8	CTGCTACCGGTTCCAATTCYCAGRCTGTGGTGACYCAG
	Reverse 3' to 5'
3' XhoI Cλ	CTCCTCACTCGAGGGYGGGAACAGAGTG
1st PCR κ LC	Forward 5' to 3'
5' L Vκ 1/2	ATGAGGSTCCCYGCTCAGCTGCTGG
5' L Vκ 3	CTCTTCCTCCTGCTACTCTGGCTCCCAG
5' L Vκ 4	ATTTCTCTGTTGCTCTGGATCTCTG
	Reverse 3' to 5'
3' Cκ 543	GTTTCTCGTAGTCTGCTTTGCTCA
2nd PCR k LC	Forward 5' to 3'
5' Pan Vκ	ATGACCCAGWCTCCABYCWCCCTG
	Reverse 3' to 5'
3' Cκ 494	GTGCTGTCCTTGCTGTCCTGCT
Specific k LC	Forward 5' to 3'
5' AgeI Vκ 1-5	CTGCAACCGGTGTACATTCTGACATCCAGATGACCCAGTC
5' AgeI Vκ 1-9	TTGTGCTGCAACCGGTGTACATTCAGACATCCAGTTGACCCAGTCT
5' AgeI Vκ 1D-43	CTGCAACCGGTGTACATTGTGCCATCCGGATGACCCAGTC
5' AgeI Vκ 2-24	CTGCAACCGGTGTACATGGGGATATTGTGATGACCCAGAC
5' AgeI Vκ 2-28	CTGCAACCGGTGTACATGGGGATATTGTGATGACTCAGTC
5' AgeI Vκ 2-30	CTGCAACCGGTGTACATGGGGATGTTGTGATGACTCAGTC
5' Age Vκ 3-11	TTGTGCTGCAACCGGTGTACATTCAGAAATTGTGTTGACACAGTC
5' Age Vκ 3-15	CTGCAACCGGTGTACATTCAGAAATAGTGATGACGCAGTC
5' Age Vκ 3-20	TTGTGCTGCAACCGGTGTACATTCAGAAATTGTGTTGACGCAGTCT
5' Age Vκ 4-1	CTGCAACCGGTGTACATTCGGACATCGTGATGACCCAGTC
	Reverse 3' to 5'
3' BsiWI Jκ 1/4	GCCACCGTACGTTTGATYTCCACCTTGGTC
3' BsiWI Jκ 2	GCCACCGTACGTTTGATCTCCAGCTTGGTC
3' BsiWI Jκ 3	GCCACCGTACGTTTGATATCCACTTTGGTC
3' BsiWI Jκ 5	GCCACCGTACGTTTAATCTCCAGTCGTGTC

Table S2. List of citrullinated peptide and protein antigens in the Luminex assay

Peptide antigens	Protein antigens
Fibrinogen A (211-230) cit cyclic	Fibrinogen cit
Fibrinogen B (246-267) cit	Vimentin cit
H2B/a (62-81) cit cyclic	Histone 2A cit
Fibrinogen A (582-599) cit	Histone 2B cit
Clusterin (221-240) cit cyclic	Apolipoprotein E cit
Biglycan (247-266) cit cyclic	CCP
H2A/a (1-20) cit cyclic	
Enolase 1A (5-21) cit	
Fibrinogen A (616-635) cit cyclic	
Vimentin (58-77) cit cyclic	
Filaggrin (48-65) cit cyclic	
Fibrinogen A (556-575) cit cyclic	
Clusterin (231-250) cit cyclic	
Apolipoprotein E (277-296) cit cyclic	

Table S3. List of clones and primers used in the overlap PCR.

CLONE	PRIMER	SEQUENCES (5'-3') *
**RA015-11_G58**	G58M38 VH Rev-F	TGAGGACACGGCTGTGTATTACTGT
	G58M38 VH Rev-R	ACAGTAATACACAGCCGTGTCCTCA
**RA015-11_K58**	K58 JH Rev-F	ACTGGCCTCAGAGTACGTTCGGCCAA
	K58 JH Rev-R	TTGGCCGAACGTACTCTGAGGCCAGT
	K58 VH Rev-F	TGCAGTCTGAAGATTTTGCAGTTTATTA
	K58 VH Rev-R	TAATAAACTGCAAAATCTTCAGACTGCA
**RA015-11_M88**	M88 VH-Rev-F	ACCGCCGCCGACACGGCCGTGTATTACTGT
	M88 VH-Rev-R	ACAGTAATACACGGCCGTGTCCGCGGCGGT
**RA015-11_K88**	K88 VH-Rev-F	ACCTGAAGATTTTGCAACTTACTACTGT
	K88 VH-Rev-R	ACAGTAGTAAGTTGCAAAATCTTCAGGT
**RA015-11_A91**	A91 JH-Rev-F	ACACGGCTGTGTATTACTGTGCGAGA
	A91 JH-Rev-R	TCTCGCACAGTAATACACAGCCGTGT
**RA015-11_K91**	K91 VH-Rev-F	AGCCTGAAGATATTGCAACATATTACTGT
	K91 VH-Rev-R	ACAGTAATATGTTGCAATATCTTCAGGCT
**RA056-11_G29**	G29 JH Rev-F	AGTGGCTTTTGACTACTGGGGCCA
	G29 JH Rev-R	TGGCCCCAGTAGTCAAAAGCCACT
	G29 VH Rev-F	TGAGGACACGGCCGTGTATTAGACA
	G29 VH Rev-R	TGTCTAATACACGGCCGTGTCCTCA
**RA056-11_KC29**	KC29 JH Rev-F	TGGGACCAAAGTGGATATCAAACGAA
	KC29 JH Rev-R	TTCGTTTGATATCCACTTTGGTCCCA
	KC29 VH Rev-F	TGAAGATTTTGCAGTGTATTACTGTCAGC
	KC29 VH Rev-R	CGTGACAGTAATACACTGCAAAATCTTCA
**RA056-11G66**	11G66-VHRevF	TGACCGCCGCAGACACGGCTGTGTATTA
	11G66-VHRevR	TAATACACAGCCGTGTCTGCGGCGGTCA
**RA056-11LC66**	11LC66JH-F	TACACTGTGGTATTCGGCGGAGGGACCA
	11LC66JH-R	TGGTCCCTCCGCCGAATACCACAGTGTA
	11LC66-VHRevF	AGGCTGAGGATGAGGCTGATTATTACT
	11LC66-VHRevR	AGTAATAATCAGCCTCATCCTCAGCCT

* In red reverted base to GL

Table S4: Ig VH and VL gene repertoire analysis of CD19+ single B cells from 3 RA synovial tissues. Clones highlighted in grey have been expressed as recombinant monoclonal antibodies

RA015/11	VH	D	JH	(-)	CDR3(aa)	(+)	Length	κ/λ	Vκ/Vλ	Jκ/Jλ	(-)	CDR3	(+)	Length
μ														
88	4-4	3-10	2	2	EVPTPYFDL	0	9	κ	1-39	2	0	QQSYSTPYT	0	9
94	1-3	4-17	3	4	GGEDGYGDSYNAFDL	0	15	κ	1-5	1	0	QQYNSYSWT	0	9
γ														
58	3-64	1-26	5	1	EIVGANRWVPVGP	1	13	κ	3-15	1	0	QQYNNWPQST	0	10
68	4-31	6-25	6	2	AISWADGYYMDV	0	12	λ	1-40	3	1	QSYDSSLSGSV	0	11
69	1-24	5-5	6	1	ASSPFYYFYSMDV	0	13	λ	2-23	3	0	CSSAGSSAVV	0	10
96	3-30	1-1	5	3	DPQNENWSPPYNWFDP	0	16	κ	1-39	2	0	QQSHSIPYT	1	9
α														
48	3-66	3-3	4	3	EGDLWSGSIDY	0	11	λ	1-44	1	2	AAWDDSLNGRV	1	11
63	4-34	3-3	5	2	APQRFVEWLFWFDP	1	14	κ	1-39	2	0	QQGYTALYN	0	9
74	4-59	5-24	6	1	GSAGGGYYYYYGMDV	0	15	λ	1-40	2/3	1	QSYDSSLSGSGV	0	12
81	1-2	4-17	3	1	GAYGDPLHI	1	9	λ	3-21	1	3	QVWDSSFDRPD	1	11
82	1-3	2-2	4	3	DRDIVVVPTARSLGYSYGSFDS	2	22	κ	1-39	1	0	QQSYTNPRT	1	9
83	4-4	2-21	3	2	GPGTATSEAFDI	0	12	λ	2-23	3	0	CSHVIGGIWV	1	10
91	3-21	3-10	4	1	WRAGVPSYFDY	1	11	κ	1-33	3	0	QQYANVFT	0	8
95	1-58	3-16	4	1	GGSYVDY	0	7	κ	4-1	2	0	QQYYSNPYT	0	9
RA015/11	VH	D	JH	(-)	CDR3(aa)	(+)	Length	κ/λ	Vκ/Vλ	Jκ/Jλ	(-)	CDR3	(+)	Length
α														
11	1-3	2-2	4	3	DRDIVVVPTARSLGYSYGSFDS	2	22							
17	3-23	3-3	4	4	SPTDFWDDYLYYFDS	0	15	κ	3-20	1	0	QQYGSSPWT	0	9
53	1-2	3-10	5	2	SEGFTHYFDP	1	10	κ	3-15	1	1	QQYNDWPQT	0	9
62	3-48	6-25	4	2	EGHNSGYDY	1	9	κ	3-11	3	0	QQRYHWPPFT	2	10
64	3-9	3-22	4	3	DISSYDDTSGYYYN	0	14	κ	3-11	1	0	QQRSNWPGT	1	9
66	4-4	6-6	3	3	KGTYSTDSYDGFDI	1	14	λ	2-23	1	0	CSYAGSSTLYV	0	11
72	1-69	3-10	5	2	ELLRGDVVPAF	1	11							
75	3-33	3-16	4	2	DVGITGMINTFDY	0	13							
80	3-9	2-8	5	2	DISRGGSSAVFEF	1	13							
86	3-23	5-24	4	2	GDGYNPGVFFDY	0	12							
88	3-11	2-2	5	1	QPWGSTNWFDP	0	11							
91	3-11	2-2	5	1	QPWGSTNWFDP	0	11							
95								λ	1-44	3	2	ASWDDSLNVWV	0	11
μ														
3	1-18	6-6	4	3	DDSYSSSFDY	0	10							
9	3-23	4-17	6	1	GQSHSWSAMDV	1	11	κ	1-17	4	0	LQHNSYPLT	1	9
12	3-15	2-21	5	2	HFESCGGDCSNW	1	12	λ	3-21	2/3	2	QVWDSSSDHPGV	1	12
19	1-2	5-5	6	3	VGGGRQLWLKDNYDYFYMDV	2	20	κ	3-20	2	0	QQYGSSHT	1	8
22	4-59	2-2	2	1	IPAAPSYWYFDL	0	12							
23	3-11	6-19	4	1	SGQQWPWDY	0	9							
70	3-23	6-19	4	1	LAFVAATWRGPFDS	1	14	κ	1-16	4	0	QQYYGYPPT	0	9
74								κ	3-15	2	0	QQYYRWPPYT	1	10
78	3-30	2-21	6	3	ELGFPYCGGDCFSSMDV	0	17							
83	3-48	6-13	4	2	DMPHFLYSSRWYPFDY	2	16	κ	1-9	4	0	QQLNSYPLT	0	9
87	5-51	5-5	4	3	AYGYIWENSRYPENFDY	1	17	κ	4-1	4	0	QQYYTTPLT	0	9
89	3-11	3-9	4	2	AYSDILTGSPYDY	0	13	κ	3-15	4	0	QQYNNWPPLT	0	10
90	4-4	2-15	4	1	LTSKLGYCSGGSCYPYFDY	1	19	λ	1-40	1	1	QSYDSSLSGSYV	0	12
92	1-2	3-22	3	3	AGYYYDSSGYLPDAFDI	0	17	λ	1-40	1	1	QSYDSSLSGSHYV	1	13

RA056/11	VH	D	JH	(-)	CDR3(aa)	(+)	Length	κ/λ	Vκ/Vλ	Jκ/Jλ	(-)	CDR3	(+)	Length
α														
23	4-59	6-19	5	2	LIAVAGTSDWFDP	0	13	λ	1-44	3	2	AAWDDSLNGPV	0	11
41								κ	3-15	1	0	QQCNNWPLT	0	9
70								λ	3-25	1	1	QSADSSGTHV	1	10
95	3-23	3-9	5	1	VPHQLVPIWFDP	1	12	λ	2-14	1	0	NSISSTSTNNV	0	11
96	3-30-3	5-24	3	2	DARGVRNAFDL	2	11	κ	1-17	1	0	LQHNSFPWT	1	9
								λ	7-43	3	1	LLYYDGGLWV	0	10
γ														
29	1-8	3-3	4	1	AAGVGVALDY	0	10	κ	3-20	3	1	QHYESSPPVFT	1	11
33	3-15	2-21	5	2	HFESCGGDCSNW	1	12	λ	4-69	1	1	QTWDTGIQV	0	9
35	3-48	3-22	4	3	VHMYYYDSSGYYYDDY	1	16	λ	2-8	1	0	SSYAGSNNYV	0	10
45	3-30	2-2	3	2	PHRLLDSCSSTSCYVVAFDL	2	20	λ	4-69	1	1	QTWDTGIQV	0	9
56	3-23	6-19	4	1	GTLSGFATTFDY	0	12	λ	2-14	1	0	SSYTSSSSLLYV	0	12
60								λ	1-44	3	2	AAWDDSLNGPV	0	11
66	4-39	1-26	4	1	RHIGRHYYFDY	4	11	λ	2-11	3/2	0	CSYVGSYTVA	0	10
68	4-61	6-6	6	2	DASIAARPPWGMDV	1	14	λ	2-23	3	0	CSYAAGNTRV	1	10
72	3-23	4-4	4	0	GARPSPSNYLYY	1	12	λ	1-44	3	2	AAWDDSLNGPV	0	11
76	1-69	3-3	5	2	VRITIFGVVMVKSDNWFDP	2	19	κ	3-15	2	2	QQYNNLYT	3	9
78	4-4	6-13	3	3	KGTYSTDSYDGFDI	1	14							
80	3-48	3-22	4	3	VHLYYYDSSGYYYDDY	1	16	λ	2-14	3/2	0	SSYTSSSTVV	0	10
83	3-9	3-22	4	3	DISSYDDTSGYYYN	0	14	λ	1-51	3/2	1	GTWDSSLSAVV	0	11
87								κ	3-15	4	0	QQYNAWPLIT	0	10
								λ	1-44	3	2	AAWDDSLNGPV	0	11
91	3-9	3-22	4	3	DISSYDDTSGYYYN	0	14	κ/λ	3-20	4	0	QQYGSSPLT	0	9
μ														
54	5-51	3-22	4	2	RYYYDSSGYTTFDY	1	14							
89	3-15	3-3	4	2	LGGYYDFWSGYYRIDY	1	16							
90	3-33	3-3	6	3	EAPIYDFWSGYRPYYYYYMDV	1	21							

RA056/11	VH	D	JH	(-)	CDR3(aa)	(+)	Length	κ/λ	Vκ/Vλ	Jκ/Jλ	(-)	CDR3	(+)	Length
α														
35														
58	1-3	2-2	4	0	SLYCSTHSCSFLHLY	2	15	κ	3-20	2	0	QQYGSSPGT	0	9
93	4-4	3-3	4	1	TFWSGSYSRYFDS	1	13	κ	3-15	5	0	QQYYNWPPIT	0	10
γ														
5	3-9	3-3	4	3	DISSYDDTSGYYYN	0	14							
6	4-31	3-10	4	2	AVNVLLRFGELRHYFDQ	3	17	κ	3-20	5	0	QQYGSLPLI	0	9
12	4-59	4-17	4	2	YGVDYFDY	0	8	λ	1-51	2/3	1	GTWDSSLSAVV	0	11
20	5-51	3-22	6	2	QGYYDRSPRPHYMDV	3	15	κ	3-11	1	0	QQRSNWPPT	1	9
21	3-30	2-2	4	1	ETCSPTNCYPRN	1	12	κ	3-11	4	0	QQRSNSLT	1	8
23	3-33	6-25	4	1	VTSRVVAAAGGYFDH	2	15	λ	3-9	2/3	1	QVWDISSVV	0	9
26	1-69	5-5	4	3	EGTAMVLEGFLDY	0	13	λ	1-44	3	1	AACDGSLNGHVV	1	12
27	4-4	2-15	6	1	GFVVVPSAMKQGNSRIPYYSYYMDL	2	25	λ	2-8	1	0	SSYAGSNNYV	0	10
35	3-9	3-22	4	3	DISSYDDTSGYYYN	0	14	κ	3-15	4	0	QQYSYWFT	0	8
								λ	1-51	2/3	1	GTWDSSLSAVV	0	11
36	4-39	1-26	4	1	RHIGRHYYFDY	4	11	λ	2-14	2/3	0	SSYTSSSTLV	0	10
39	3-9	3-22	4	3	DISSYDDTSGYYYN	0	14	λ	1-47	3	2	AAWDDSLSGWV	0	11
42								κ	4-1	4	0	QQYYSTLALT	0	10
45	3-30	2-2	6	2	DIVVVPAATSLLGGYYYYYMDV	0	22	κ	1-39	4	0	QQSSTTPLT	0	9
48	3-15	2-21	5	2	HFESCGGDCSNW	1	12	κ	1-39	2	0	QQSYSTPYT	0	9
54	1-46	1-7	6	3	DGLEARRTTSSHPHYYMDV	4	19	κ	3-11	4	0	QLRSNWRT	2	8
56	4-34	6-6	6	1	KKGRVGIAYMEV	3	12	κ	1-39	3	0	QQSFSMPFT	0	9
59	3-9	6-13	4	2	DSAAGTPVYFDY	0	12	κ	1-33	4	1	QQYDNLPLT	0	9
								λ	1-44	3	2	AAWDDSLNGPV	0	11
63	5-51	3-3	5	1	HRGPTTIFGVAIGAFDP	2	17	λ	1-44	3	2	AVWDDSLNGPV	0	11
66	-30-4(4-31	3-9	5	3	GVFDNFFNRRLETNWFDP	2	18	κ	3-11	3	0	QQRSSWPPT	1	9
72	3-15	2-21	5/4	2	HFESCGGDCSNW	1	12	λ	6-57	2/3	2	QSYDNDNLWV	0	10
75	4-59	5-5	2	2	TPYPPLDWYFDL	0	12	κ	4-1	1	0	QQYYITPPT	0	9
88	3-15	2-21	5/4	2	HFESCGGDCSNW	1	12	λ	1-44	1	2	AAWDDSLNGYV	0	11
94	3-30	3-10	4	3	EVREYTDY	1	8	κ	3-11	4	0	QLRSNWLLT	1	9
95	3-23	1-26	4/3	0	LVGITHLSAAPWT	1	13	λ	2-23	3	0	CSYAGTWV	0	8
μ														
2								λ	1-44	3	2	AAWDDSLNGPV	0	11
3	3-21	3-22	3	3	DYYDSSGYLSAFDI	0	14	λ	1-44	3	2	AAWDDSLNGWV	0	11
4	1-18	4-17	4	2	ASYGDYSDY	0	9	κ	1-13	2	0	QQYYSTPPYT	0	10
								λ	1-44	3	2	AAWDDSLNGPV	0	11
9	3-23	3-10	3	4	CETGERRWYYYGSGTIREAFDI	3	22	κ	3-11	4	0	QQRSNWPPT	1	9
15	1-69	4-17	4	1	DQATTVTTRVWFVY	1	14	λ	1-47	3	2	AAWDDSLSGRV	1	11
24	1-3	2-2	4	0	VGIVVVPAAALGY	0	13							
25								λ	1-44	3	2	AAWDDSLNGPV	0	11
34	3-21	6-13	5	3	PRQLGSVWFDP	4	12	κ	1-9	4	0	QQLNSYPLT	0	9
38	3-33	6-13	4	2	DRSSSWYFDH	2	10	λ	2-14	3	0	SSYTSSSTWV	0	10
39								λ	1-47	3	2	AAWDDSLSGWV	0	11
41	3-23	3-10	4	1	GSGTFDY	0	7	λ	3-21	1	2	QVWDSSSDHYV	1	11
48	4-31	6-6	6	1	VSLNSSSSLIHYYYYMDV	1	18	κ	1-39	2	0	QQSYSTPYT	0	9
67	3-30	5-12	6	3	DYTAWLLNEYYYYGMDV	0	17	λ	1-47	3	2	AAWDDSLSVWV	0	11
81	3-64	3-3	4	3	EYDFWSGYYYRGATRTTPNFDY	2	22	κ	3-15	2	0	QQYNNWPLWT	0	10
91	1-69	5-5	4	3	DLVDPHLLTHGFDY	2	14	κ	3-15	2	0	QQYNGWPLPMYT	0	12

RA057/11	VH	D	HJ	(-)	CDR3(aa)	(+)	Length	κ/λ	Vκ/Vλ	Jκ/Jλ	(-)	CDR3	(+)	Length
α														
6	3-30	2-15	4	0	AHIVVVVAASYYFAY	1	15							
12	3-11	3-3	4	1	SFWNGYHFDY	1	10	λ	2-14	2/3	0	SSYTTSGTYVV	0	11
25	3-7	6-13	6	3	DQVEQQLVLGYFYYYYMDV	0	19	λ	1-44	1	1	AAWDASLKV	1	9
43	3-33	4-17	6	2	ADYGNSYYYYMDV	0	13	λ	2-8	1	0	SSYAGSNNYV	0	10
47	3-7	3-3	4	4	DPRAYDYWSGYYEGYFDY	1	18	κ	3-20	1	0	QQYGSSPGT	0	9
								λ	2-23	3	0	CSSASFTISWV	0	11
γ														
3	4-39	2-15	5	1	LFGPGIPGWWLDP	0	13	κ	3-20	4	0	QQHGSSRGLT	2	10
5	1-2	5-5	5	1	TSMGYTSTWAYNWFDP	0	16	λ	2-23	2	2	CLYAGEEVL	0	9
10	4-34	5-5	6	2	GVRGGYTSDSFPLYYFMDL	1	19	κ	1-12	5	0	QQANSFPVT	0	9
26	3-21	1-14	3	1	TPNWNHRGGAFDI	2	13							
29	1-69	3-16	6	1	GRLPWRYMDV	2	10							
31	1-18	2-15	6	1	TPRYYYYYMDV	1	11							
36	4-28	2-2	6	1	RALYHHYMDV	3	10	κ	3-20	2	0	QQHGSSPYT	1	9
								λ	2-8	2/3	0	SSYAGVHTVI	1	10
47	3-48	3-22	4	3	VHLYYYDSSGYYYDDY	1	16	κ/λ	3-20	1	0	QQYGSSPGT	0	9
50	3-9	3-16	6	1	GSYRYYYYCIDV	1	12	κ	3-20	2	0	QQYGSSPVYS	0	10
52	4-31	3-3	3	2	LVGIFGGDAFDI	0	12	λ	1-51	2/3	2	EVWDSGLSVRL	1	11
57	3-48	6-19	4	1	AGLSGSGPFDY	0	11	λ	1-47	2/3	2	AAWDDSLSGVV	0	11
68	3-30	6-19	4	1	PAVVGASLHFDY	1	12	κ/λ	3-20	2	0	QQHGSSPYT	1	9
69								κ	1-27	1	0	QKYNSAPRT	2	9
72	3-23	1-26	5	1	HWDS	1	4	λ	1-44	1	1	SAWDNSLNGYF	0	11
77	3-72	1-7	6	2	VKATYGWKINVDQFLDV	2	17	κ/λ	1-39	1	0	QQSFRTPWT	1	9
78	3-21	3-3	4	1	LGYDFWSGHRH	3	11	λ	6-57	1	2	WSYDNYQEI	0	9
80	3-48	3-22	4	3	VHLYYYDSSGYYYDDY	1	16	κ	3-20	1	0	QQYGTSPWT	0	9
								λ	1-47	2/3	2	AAWDDSLSGVV	0	11
89	4-59	??	2	2	TPYPPLDWYFDL	0	12	κ/λ	1-47	2/3	2	AAWDDSLSGVV	0	11
91	3-48	3-22	4	3	VHLYYYDSSGYYYDDY	1	16	κ	3-20	2	0	QQHGSSPYT	1	9
								λ	2-14	2/3	0	SSYTSSSTRRVV	2	12
93	3-74	3-3	6	5	DGGEAYDFWSDNHRFYFYYYMDV	2	23	λ	2-14	2/3	1	SSYTTSSDLV	0	10
μ														
2	1-46	4-23	4	3	FGRHDYGGKDDY	3	12	κ	1-33	2	1	QQYDNLPYT	0	9
17	4-31	3-10	6	3	DQITMVRGGDGQNYYYYYMDV	1	21	κ/λ	1-39	3	0	QQSYSTPPLST	0	11
20	3-9	6-19	4	2	DSRRKSIAVAGAYFDY	3	16	κ/λ	2-8	1	0	SSYAGSNNYV	0	10
21	3-23	3-9	6	3	DGRPSTIFWDYMDV	1	14	λ	2-14	2	0	*RSYTTINNLST*	1	11
28	3-21	2-21	4	3	DVGDIVVVTASLDY	0	14	λ	1-44	2	2	AAWDDSLNGVV	0	11
29	3-23	5-5	3	1	GIQLWPGGAFDI	0	12							
30	3-30	3-10	4	2	DLAPGFTSAFDY	0	12	κ	4-1	4	0	QQYYTLPLT	0	9
32	3-23	3-22	4	3	DGYYDRRGGTVDY	2	13	κ	3-15	1	0	QQYNNWPPWT	0	10
								λ	2-8	1	0	SSYAGSNNYV	0	10
35	4-34	6-13	4	1	GWAYSSSWYRRMISFDY	2	17	κ/λ	3-20	2	0	QQHGSSPYT	1	9
44	1-46	3-22	4	2	VGGGYYDSSGGALDY	0	15	κ	1-5	1	0	QQYNSYPWT	0	9
48	3-23	5-12	4	2	VGYSGYDLRVYYFDY	1	15	λ	2-8	1	0	SSYAGSNNYV	0	10
51	4-59	2-21	3	2	RVGSPYCGGDCYPAFDI	1	17	κ	1-8	3	0	QQYYSYPT	0	8
56	5-51	2-2	6	2	ILVDCSSTSCYYYYYYMDV	0	19	λ	3-25	2	1	QSADSSGLV	0	9
58	3-30	1-7	4	1	QWGATGTLDY	0	10	κ	4-1	3	0	QQYYSTPT	0	8
61	3-21	6-13	4	1	GGSSWYYFDY	0	10	λ	1-44	3	2	AAWDDSLNGWV	0	11
62	3-7	3-3	4	1	ELFHILSY	1	8	κ	1-33	2	1	QQYDNLPLT	0	9
								λ	1-44	2/3	2	AAWDDSLNGPV	0	11
63	3-11	6-13	6	2	EMGGSSWSIYYYYYYMDV	0	18	κ	1-5	2	0	QQYNSSPYT	0	9
								λ	1-47	2/3	2	AAWDDSLSGVV	0	11
67	4-59	7-27	3	2	RESSRLGNAFDI	2	12	λ	2-23	2/3	0	CSYAGSSTL	0	9
71	1-18	5-24	4	2	DLNSYYFDY	0	9	κ	4-1	4	0	QQYYSTPLT	0	9
								λ	2-8	1	0	SSYAGSNNYV	0	10
75	3-21	1-7	2	1	AGNWNYYWYFDL	0	12	λ	2-8	1	0	SSYAGSNNYV	0	10
82	1-24	1-26	3	1	PIVLGAFDI	0	9	κ	3-20	2	0	QQYGSSPPYT	0	10
								λ	2-23	2	0	CSYAGSPV	0	8
89	1-18	2-2	6	1	RYCSSTSCYKGSYYYYYYYMDV	2	22	κ	3-20	4	0	QQYGSSPLT	0	9
								λ	1-47	2/3	2	AAWDDSLSGVV	0	11
